# A network meta-analysis of interventions for anxiety and depression in PCOS

**DOI:** 10.7717/peerj.20744

**Published:** 2026-02-05

**Authors:** Zuolin Tan, Yunqing Li, Jingyuan Liu, Xinyin Hu, Xuhan Su, Yuhua Huang

**Affiliations:** 1 Beijing University of Chinese Medicine, Beijing, China; 2 Capital Medical University, Beijing Hospital of Traditional Chinese Medicine, Beijing, China

**Keywords:** Polycystic ovary syndrome, Anxiety, Depression, Systematic review, Network meta-analysis

## Abstract

**Background:**

The study aimed to provide evidence to support optimal interventions for alleviating anxiety and depression symptoms in patients with polycystic ovarian syndrome (PCOS) through a systematic review and network meta-analysis.

**Methods:**

A comprehensive literature search of PubMed, Embase, Cochrane Library, and Web of Science from their inceptions to January 2, 2025 was performed. The criteria for inclusion defined were as follows: (1) The study population consisted of female PCOS patients; (2) interventions included psychological therapy, exercise, drug treatment, or digital intervention; (3) studies that reported changes in anxiety and depression scores; and (4) randomized controlled trials (RCTs). Two reviewers independently screened the literature and extracted the data. Disagreements were resolved by consulting a third party. Standardized mean difference (SMD) was used for data recording in this study. The analysis of data was carried out based on a random-effects model, while network meta-analysis was implemented through R 4.4.0 and Just Another Gibbs Sampler (JAGS) 4.3.1. We conducted a Bayesian random-effects network meta-analysis (NMA) and ranked interventions using the surface under the cumulative ranking curve (SUCRA).

**Results:**

This study included a total of 25 RCTs, involving 1,453 female PCOS patients, to evaluate the effects of various interventions in alleviating anxiety and depression symptoms. Effective interventions included emotion-focused therapy (EFT), peer support (PS), omega-3 plus vitamin E (O3+VE), and mindfulness stress management (MSM). Other interventions, such as metformin and vitamin D plus probiotics (VD+Pro), showed no significant benefit compared with control. Data on PS for anxiety were not analyzed in the present network meta-analysis because relevant trials could not be connected within the network structure.

**Conclusion:**

Our study demonstrates that EFT and PS emerge as promising interventions in alleviating anxiety and depression symptoms in PCOS patients. Interventions such as O3+VE and MSM also showed potential in improving emotional states.

**Review registration:** PROSPERO CRD420250655513.

## Introduction

Polycystic ovary syndrome (PCOS) is a common endocrine disorder affecting about 10% of females of childbearing potential worldwide (Goodarzi et al., 2011; Lizneva et al., 2016; Stener-Victorin et al., 2024). The main characteristics of PCOS include ovulatory dysfunction, hyperandrogenism, and polycystic ovarian morphology. These physiological symptoms not only impact women’s reproductive health but are also associated with various metabolic abnormalities, including obesity, insulin resistance, type 2 diabetes mellitus, and cardiovascular diseases (Patel, 2018; Stener-Victorin et al., 2024)

PCOS patients experience significantly higher rates of anxiety and depression than the general population, contributing factors include hormonal fluctuations, changes in physical appearance (such as weight gain and hirsutism), fertility issues, and the long-term management burden of chronic conditions (Shrivastava & Conigliaro, 2023). Neuroendocrine dysregulation, such as hyperandrogenism and insulin resistance, may disrupt the hypothalamic–pituitary–adrenal (HPA) axis and neurotransmitter balance, thereby predisposing patients to mood disturbances (Ethirajulu et al., 2021). Chronic stress and inflammatory responses further amplify this feedback loop, increasing vulnerability to anxiety and depression (Hassamal, 2023). These factors collectively contribute to the increased psychological health risks faced by these patients. In addition, anxiety and depression can exacerbate the physiological symptoms of PCOS, leading to issues such as sleep disturbances, eating disorders, and impaired social functioning, which further negatively impact the overall health of patients (Rodriguez-Paris et al., 2019). Therefore, effectively addressing mental health issues in affected patients has become an integral component of comprehensive treatment.

Currently, there are various interventions targeting anxiety and depression symptoms in PCOS patients, encompassing drug treatment, lifestyle interventions, and psychological therapies, and digital approaches. Pharmacological treatments, including oral contraceptives, insulin sensitizers, and anti-androgen medications are widely used to regulate menstrual cycles, improve metabolic parameters, and reduce androgen levels (Sadeghi et al., 2022). Lifestyle interventions primarily consist of dietary adjustments and physical exercise. Among exercise interventions, moderate-intensity continuous training (MICT) and high-intensity interval training (HIIT) have been proven to improve the mental health of PCOS patients by alleviating anxiety and depression symptoms (Patten et al., 2023). Psychological therapies, such as cognitive-behavioral therapy (CBT) and emotion-focused therapy (EFT), have been widely applied in the intervention of various psychological issues and have shown promising effects (Amirshahi et al., 2024). In addition, digital interventions, such as mobile applications and online psychological therapy platforms, are emerging as novel treatment approaches (Wang et al., 2022). Due to their convenience and low cost, these interventions are increasingly favored by many patients. However, the effects of these therapeutic approaches on anxiety and depression symptoms in PCOS patients have not yet been systematically evaluated.

To comprehensively study the impact of different interventions on anxiety and depression in PCOS patients, this study employed systematic review and network meta-analysis for investigation. Network meta-analysis allows both direct and indirect comparisons across multiple interventions, providing a more comprehensive evidence base (García-Hermoso et al., 2023; Hutton et al., 2015). Previous meta-analyses have examined narrower intervention categories, such as cognitive behavioural therapy (CBT) for depression in PCOS (Jiskoot et al., 2022) and mind–body interventions focusing mainly on quality of life and depression outcomes (Zhao et al., 2024). While these reviews provided valuable insights, they were limited to specific intervention types and outcomes. This approach can thoroughly compare and assess the effects of various interventions in improving anxiety and depression. Such an analytical method provides high-quality and evidence-based support for clinical decision-making, thereby facilitating clinicians to select the most appropriate treatment regimen based on the specific circumstances of individual patients.

## Methods

This study adhered to the Preferred Reporting Items for Systematic Reviews and Meta-Analyses (PRISMA), including those specifically for network meta-analysis (Hutton et al., 2015; Page et al., 2021). Study design and implementation commenced on 20 December 2024, and this study’s protocol was registered with PROSPERO (ID: CRD420250655513) on 24 February 2025.

### Literature search

This study utilized multiple databases for literature search, including PubMed, Embase, Cochrane, and Web of Science, with the search date range spanning from database inception to January 2, 2025. The primary search keywords included “polycystic ovary syndrome”, “anxiety”, and “depression”, and a comprehensive search term library was developed by combining subject headings and free-text terms. Additionally, Boolean operators were employed to formulate the search strategy to ensure all relevant literature was covered (detailed search strategies are presented in Table S1).

### Screening of literature and eligibility criteria

The screening process was independently conducted by two researchers. First, duplicate entries in the search results were removed using EndNote 21 to ensure each literature appeared only once. Next, studies that failed to meet the eligibility criteria were excluded depending on their type. Following the initial screening, the researchers independently examined the titles and abstracts of these articles to determine which studies might meet the criteria for inclusion. For these potentially eligible studies, the full texts were downloaded and reviewed to confirm whether they met the defined requirements. In cases where the opinions of the two researchers differed, a third researcher participated in the evaluation to ensure objectivity and consistency in the screening process. Eligibility criteria are as follows:

### Criteria for inclusion

Population: women diagnosed with PCOS; interventions: including but not limited to health education, mood management, exercise, nutritional supplements, or other drug interventions, without specific restrictions; comparator: including comparisons between different interventions, or using a placebo control; outcome: changes in anxiety and depression scores, using relevant scales to measure changes before and after intervention; study design: randomized controlled trial (RCT).

Criteria for exclusion: (1) Withdrawn articles; (2) study with a total sample size of less than 20; (3) there is overlap in samples in the studies, and studies with larger sample sizes were preferred; (4) lack of anxiety or depression score data or unable to calculate its changes; (5) no single intervention could be considered for inclusion in the network meta-analysis; (6) letters to the editor, comments, reviews, editorials, and expert opinions; (7) case series reports, case reports, conference abstracts, and animal experiments; (8) literature not in English.

### Quality evaluation of included studies

Two reviewers independently appraised the quality of all included studies with the National Institutes of Health (NIH) quality evaluation tool for RCTs (National Institutes of Health, 2021). This tool consists of 14 evaluation items, with each item rated as “Yes” or “No” based on the standards of study design and execution, and corresponding scores assigned (“Yes” scores 1 point, “No” scores 0 points). When scoring discrepancies arose between the two reviewers, a third reviewer was consulted for settlement to ensure fairness and consistency in the evaluation. Under predefined criteria, all studies were categorized for risk of bias by reviewers: studies scoring 0–5 were classified as high risk of bias (Poor), those scoring 6-10 as moderate risk of bias (Fair), and those scoring 11–14 as low risk of bias (Good).

### Data extraction

The following data was extracted independently and in duplicate by two reviewers (Zuolin Tan and Yunqing Li): the fundamental study information, such as first author, publication year, sample size, and country of origin; the basic demographic characteristics of the patients, such as age, sex, intervention measures, and the duration of the intervention; the key outcome data, including the average anxiety or depression scores along with their respective standard deviations before and after the intervention, or the mean and standard deviation of score changes before and after the intervention. Two reviewers independently extracted and organized all data into tables. Disagreements between the two reviewers were documented and resolved either through discussion with a third reviewer (Xinyin Hu) or by contacting the authors for clarification.In cases of data discrepancies, they were resolved through discussion to ensure that the data were accurate and reliable.

### Data collection and statistical analysis

Data analysis was conducted with R 4.4.0 (R Core Team, 2024) and Just Another Gibbs Sampler (JAGS) 4.3.1. Because included trials used different anxiety and depression scales, we analyzed standardized mean differences under a random-effects model. To ensure a consistent direction of effect, we harmonized all scales so that higher scores indicated worse symptoms; thus, negative change values reflected improvement. The primary metrics used to evaluate treatment efficacy and safety were the standardized mean difference (SMD) and 95% credible intervals (CrIs), which provide a Bayesian probability-based interval estimate analogous to traditional confidence intervals (CIs). As SMD represents standardized relative differences across scales, it reflects the magnitude of effect rather than absolute score changes on a given instrument. The results of the analysis were presented using forest plots, league tables, and the surface under the cumulative ranking curve (SUCRA) values, where greater SUCRA values signify superior ranking for the intervention. In addition, for the two outcome measures of anxiety and depression, SUCRA cluster analysis plots were generated. Interventions positioned nearer to the top-right corner indicate better intervention effects. When league-table comparisons show overlapping 95% CrIs, SUCRA ranks are considered indicative rather than definitive. Given network sparsity for several interventions, we did not perform subgroup or additional sensitivity analyses to avoid fragmentation of evidence and potential network disconnection; uncertainty is reflected in the CrIs of the primary random-effects model.

## Results

### Literature retrieval

**Figure 1:**
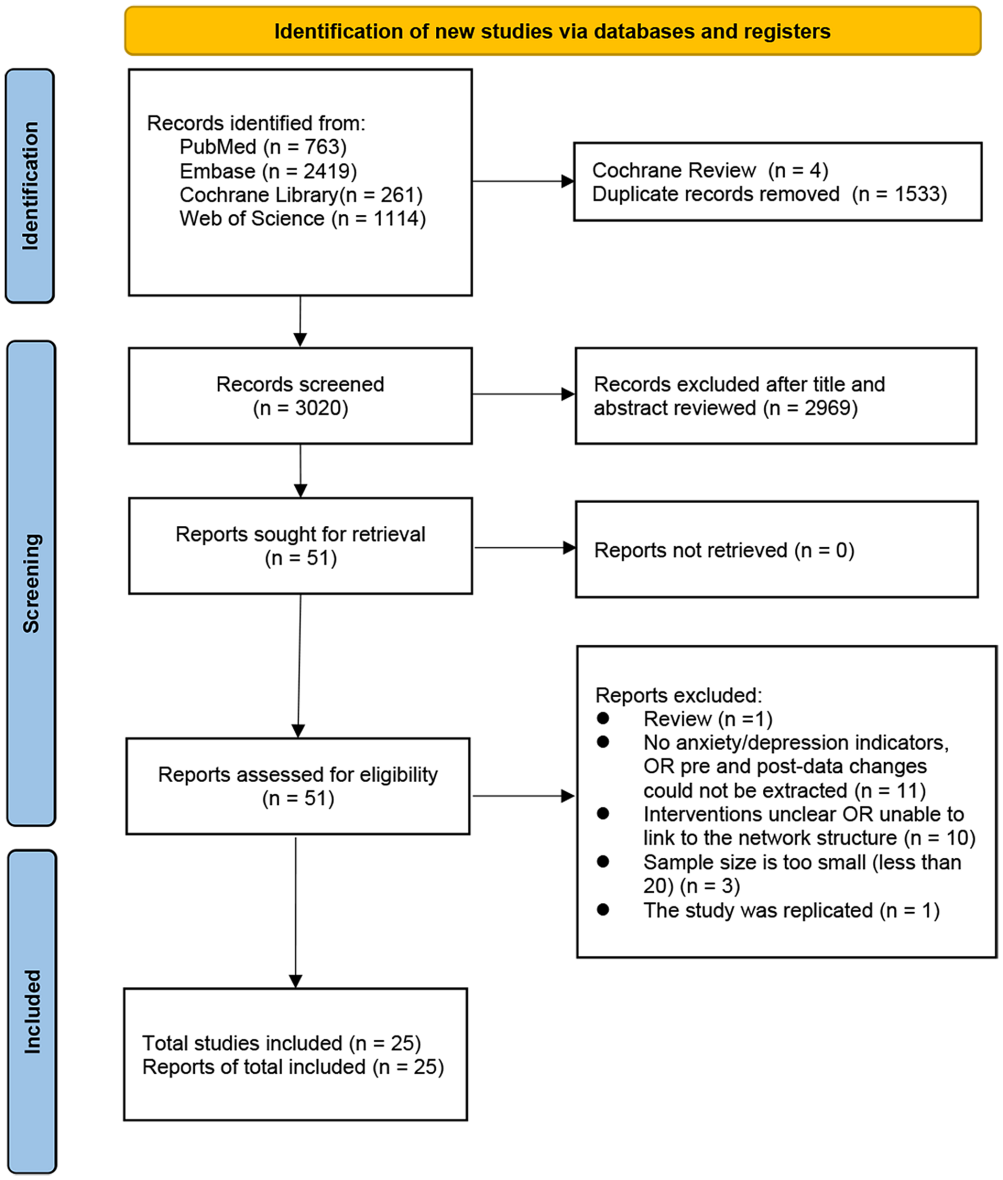
PRISMA flowchart for screening of literature.

We identified a total of 4,557 articles. After excluding 4 Cochrane Reviews and 1,533 duplicates, 3,020 articles remained. The titles and abstracts of these articles were preliminarily reviewed and screened, resulting in the exclusion of 2,969 articles unrelated to the study topic. The remaining 51 articles were all accessible in full text. Subsequently, detailed screening of these articles was conducted based on predefined criteria, leading to the exclusion of the following: one review article, 11 studies that did not explicitly report anxiety or depression measures or for which relevant outcome measures could not be extracted, 10 studies with interventions that could not be connected within the network structure, three studies with a sample size of fewer than 20, and one duplicate study. Ultimately, 25 studies (25 articles) were included. The PRISMA flowchart for selecting the studies is presented in Fig. 1, Table S2.

### Basic information of included studies

This study included 25 RCTs involving a total of 1,453 participants, with data covering multiple countries, including Sweden, Denmark, Iran, Brazil, India, South Korea, China, Australia, Greece, Sri Lanka, and the United States. The age range of participants was 15 to 45 years, with most individuals falling between 18 and 40. Interventions included acupuncture (Acup), moderate-intensity continuous training (MICT), high-intensity interval training (HIIT), escitalopram (Esc), carnitine and chromium (Car+Chr), emotion-focused therapy (EFT), Cognitive-behavioral therapy (CBT), coenzyme Q10 (CoQ10), Vitamin K2 (VK2), digital interventions (Dig), melatonin (Mel), MIND Diet (MIND), Omega-3 + Vitamin E (O3+VE), probiotic + selenium (Pro+Sel), Vitamin D and Omega-3 (VD+O3), Vitamin D and probiotics (VD+Pro), metformin (Met), pioglitazone metformin complex (PM), mindfulness stress management (MSM), peer support (PS), myoinositol (Myo), and yoga.

For the use of scales, the evaluation of anxiety and depression primarily relied on widely used standardized tools, including the Brief Scale for Anxiety (BSA-S), the Montgomery-Åsberg Depression Rating Scale (MADRS-S), the Beck Anxiety Inventory (BAI), the Beck Depression Inventory (BDI), and the Hospital Anxiety and Depression Scale (HADS). Additionally, various other psychological health evaluation tools were employed, such as the Depression, Anxiety, and Stress Scales (DASS), the Generalized Anxiety Disorder 7-item scale (GAD-7), and the Symptom Checklist-90-Revised (SCL-90-R). Although total trial sizes were ≥20 by design, arm-level sizes varied substantially (9–53 per arm), which may limit precision and suggests that small-study bias cannot be excluded. Details are presented in Table 1.

**Table 1:** Fundamental features of the included studies.

Study	Country	Age range (years)	Intervention	Intervention abbreviation	Sample size	Duration	Anxiety scale	Depression scale
Stener-Victorin et al. (2013)	Sweden	NA	Acupuncture	Acup	28	16 weeks	BSA-S	MADRS-S
			Moderate-Intensity Continuous Training	MICT	29			
								
			Control	Ctrl	15			
Glintborg et al. (2018)	Denmark	NA	Escitalopram	Esc	20	12 weeks	NA	MDI
			Control	Ctrl	19			
Jamilian et al. (2019)	Iran	18∼40	Carnitine and chromium	Car+Chr	26	12 weeks	DASS (Anxiety)	BDI
			Control	Ctrl	27			
Amirshahi et al. (2024)	Iran	25∼45	Emotion-focused therapy	EFT	15	8 weeks	BAI	BDI
			Cognitive-behavioral therapy	CBT	15			
			Control	Ctrl	15			
Lopes et al. (2018)	Brazil	18∼39	Moderate-Intensity Continuous Training	MICT	23	16 weeks	HADS ( Anxiety)	HADS (Depression)
			High-Intensity Interval Training	HIIT	22			
			Control	Ctrl	24			
Nidhi et al. (2012)	India	15∼18	Yoga	Yoga	45	12 weeks	STAI	NA
			Moderate-Intensity Continuous Training	MICT	45			
Tarkesh et al. (2022)	Iran	18∼40	Vitamin K2	VK2	42	8 weeks	NA	BDI
			Control	Ctrl	42			
Lee & Lee (2023)	Korea	18∼40	Digital	Dig	14	12 weeks	NA	K-CESD
			Control	Ctrl	14			
Karamali & Gholizadeh (2022)	Iran	18∼40	CoQ10	CoQ10	28	12 weeks	BAI	BDI
			Control	Ctrl	27			
Wang et al. (2019)	China	18∼28	Acupuncture	Acup	27	16 weeks	Zung-SAS	Zung-SDS
			Control	Ctrl	27			
Shabani et al. (2019)	Iran	18∼40	Melatonin	Mel	29	12 weeks	BAI	BDI
			Control	Ctrl	29			
Kabiri et al. (2024)	Iran	18∼45	MIND diet	MIND	26	8 weeks	DASS (Anxiety)	DASS (Depression )
			Control	Ctrl	26			
Jamilian et al. (2018c)	Iran	18∼40	Omega - 3 + Vitamin E	O3+VE	20	12 weeks	DASS (Anxiety)	BDI
			Control	Ctrl	20			
Jamilian et al. (2018a)	Iran	18∼40	Probiotic + Selenium	Pro+Sel	30	12 weeks	DASS (Anxiety)	BDI
			Control	Ctrl	30			
Patten et al. (2023)	Australia	18∼45	High-Intensity Interval Training	HIIT	14	12 weeks	DASS (Anxiety)	DASS (Depression )
			Moderate-Intensity Continuous Training	MICT	15			
Dilimulati et al. (2024)	China	18∼40	Digital	Digital	40	12 weeks	HADS (Anxiety)	HADS (Depression)
			Metformin	Met	40			
Stefanaki et al. (2015)	Greece	15∼40	Mindfulness Stress Management	MSM	23	8 weeks	DASS (Anxiety)	DASS (Depression)
			Control	Ctrl	15			
Jamilian et al. (2018b), Ranasinghe et al. (2023)	Sri Lanka	18∼39	Peer Support	PS	20	10 weeks	NA	CESD
			Control	Ctrl	22			
Jamilian et al. (2018b)	Iran	18∼40	Vitamin D and omega - 3	VD+O3	30	12 weeks	DASS (Anxiety)	BDI
			Control	Ctrl	30			
AlHussain et al. (2020)	Saudi Arabia Jordan	NA	Metformin	Met	53	3 months	GAD-7	PHQ-9
			Control	Ctrl	33			
Ravn et al. (2022)	Denmark	18∼50	Myoinositol	Myo	22	6 months	NA	BDI
			Metformin	Met	23			
Guo et al. (2020)	China	20∼35	Control	Ctrl	21	12 weeks	SCL-90-R-A	SCL-90-R-D
			Metformin	Met	26			
			Pioglitazone Metformin Complex	PM	28			
Patel et al. (2020); Wang et al. (2022)	USA	23∼42	Yoga	Yoga	9	3 months	BAI	BDI
			Control	Ctrl	51			
Wang et al. (2022)	China	18	Digital	Dig	49	12 months	Zung-SAS	Zung-SDS
			Control	Ctrl	30			
Ostadmohammadi et al. (2019)	Iran	18∼40	Vitamin D and Probiotics	VD+Pro	30	12 weeks	DASS (Anxiety)	BDI
			Control	Ctrl	30			

**Notes.**

BSA-S Brief Scale for Anxiety MADRS-S Depression Rating Scale MDI Major Depression Inventory DASS Depression Anxiety and Stress Scale DASS (Anxiety) Depression Anxiety and Stress Scale (for Anxiety) DASS (Depression) Depression Anxiety and Stress Scale (for Depression) BAI Beck Anxiety Inventory BDI Beck Depression Inventory STAI State-Trait Anxiety Inventory HADS Hospital Anxiety and Depression Scale HADS (Anxiety) Hospital Anxiety and Depression Scale (for Anxiety) HADS (Depression) Hospital Anxiety and Depression Scale (for Depression) K-CESD Epidemiological Studies Depression Scale; SCL-90-R-A Symptom Checklist 90-R (Anxiety) SCL-90-R-D Symptom Checklist 90-R (Depression) GAD-7 Generalized Anxiety Disorder 7 PHQ-9 Patient Health Questionnaire-9

### Risk of bias evaluation

The overall quality of the studies included in the analysis was good, with most meeting high standards regarding randomization, concealment of random allocation, participants and personnel blinding, and blinded outcome evaluation. The majority of studies (15 studies) employed intention-to-treat analysis. However, while most studies reported adequate sample sizes and appropriate intergroup differences, a few studies lacked sufficient reporting on the similarity of interventions and high protocol adherence rates. Details are described in Table 2.

**Table 2:** NIH Risk of bias score.

Study	1. Randomized?	2. Randomization adequate?	3. Allocation concealed?	4. Blinded participants and providers?	5. Blinded outcome assessors?	6. Groups similar at baseline?	7. Drop-out rate <20%?	8. Differential drop-out rate < 15%?	9. High adherence to protocol?	10. Other interventions similar?	11. Valid outcome measures?	12. Sufficient sample size?	13. Prespecified outcomes?	14. Intention- to-treat analysis?	Sum (Grade)
Stener-Victorin et al. (2013)	Y	Y	Y	N	Y	Y	Y	Y	Y	Y	Y	Y	Y	Y	13 (Good)
Glintborg et al. (2018)	Y	Y	Y	Y	Y	Y	Y	Y	Y	Y	Y	Y	Y	N	13 (Good)
Jamilian et al. (2019)	Y	Y	Y	Y	Y	Y	Y	Y	Y	Y	Y	Y	Y	N	13 (Good)
Amirshahi et al. (2024)	Y	Y	Y	N	Y	Y	Y	Y	Y	Y	Y	Y	Y	Y	13 (Good)
Lopes et al. (2018)	Y	Y	Y	N	Y	Y	N	Y	Y	Y	Y	Y	Y	N	11 (Good)
Nidhi et al. (2012)	Y	Y	Y	N	Y	Y	N	Y	Y	Y	Y	Y	Y	Y	12 (Good)
Tarkesh et al. (2022)	Y	Y	Y	Y	Y	Y	Y	Y	Y	Y	Y	Y	Y	Y	14 (Good)
Lee & Lee (2023)	Y	Y	Y	N	Y	Y	Y	Y	Y	Y	Y	Y	Y	Y	13 (Good)
Karamali & Gholizadeh (2022)	Y	Y	Y	Y	Y	Y	Y	Y	Y	Y	Y	Y	Y	N	13 (Good)
Wang et al. (2019)	Y	Y	Y	Y	Y	Y	N	Y	Y	Y	Y	Y	Y	N	12 (Good)
Shabani et al. (2019)	Y	Y	Y	Y	Y	Y	Y	Y	Y	Y	Y	Y	Y	Y	14 (Good)
Kabiri et al. (2024)	Y	Y	Y	Y	Y	Y	Y	Y	Y	Y	Y	Y	Y	Y	14 (Good)
Jamilian et al. (2018c)	Y	Y	Y	Y	Y	Y	Y	Y	Y	Y	Y	Y	Y	Y	14 (Good)
Jamilian et al. (2018a)	Y	Y	Y	Y	Y	Y	Y	Y	Y	Y	Y	Y	Y	Y	14 (Good)
Patten et al. (2023)	Y	Y	Y	N	Y	Y	N	Y	Y	Y	Y	Y	Y	Y	12 (Good)
Dilimulati et al. (2024)	Y	Y	Y	Y	Y	Y	Y	Y	Y	Y	Y	Y	Y	Y	14 (Good)
Stefanaki et al. (2015)	Y	Y	Y	N	Y	Y	N	N	Y	Y	Y	Y	Y	N	10 (Fair)
Ranasinghe et al. (2023)	Y	Y	Y	N	Y	Y	N	Y	Y	Y	Y	Y	Y	N	11 (Good)
Jamilian et al. (2018b)	Y	Y	Y	Y	Y	Y	Y	Y	Y	Y	Y	Y	Y	Y	14 (Good)
AlHussain et al. (2020)	Y	Y	Y	Y	Y	Y	Y	Y	Y	Y	Y	Y	Y	Y	14 (Good)
Ravn et al. (2022)	Y	Y	Y	Y	Y	Y	N	N	Y	Y	Y	Y	Y	N	11 (Good)
Guo et al. (2020)	Y	Y	Y	Y	Y	Y	Y	Y	Y	Y	Y	Y	Y	Y	14 (Good)
Patel et al. (2020)	Y	Y	Y	N	Y	Y	N	N	Y	Y	Y	Y	Y	N	10 (Fair)
Wang et al. (2022)	Y	Y	Y	N	Y	Y	Y	Y	Y	Y	Y	Y	Y	N	12 (Good)
Ostadmohammadi et al. (2019)	Y	Y	Y	Y	Y	Y	Y	Y	Y	Y	Y	Y	Y	Y	14 (Good)

### Network meta-analysis of anxiety

**Figure 2:**
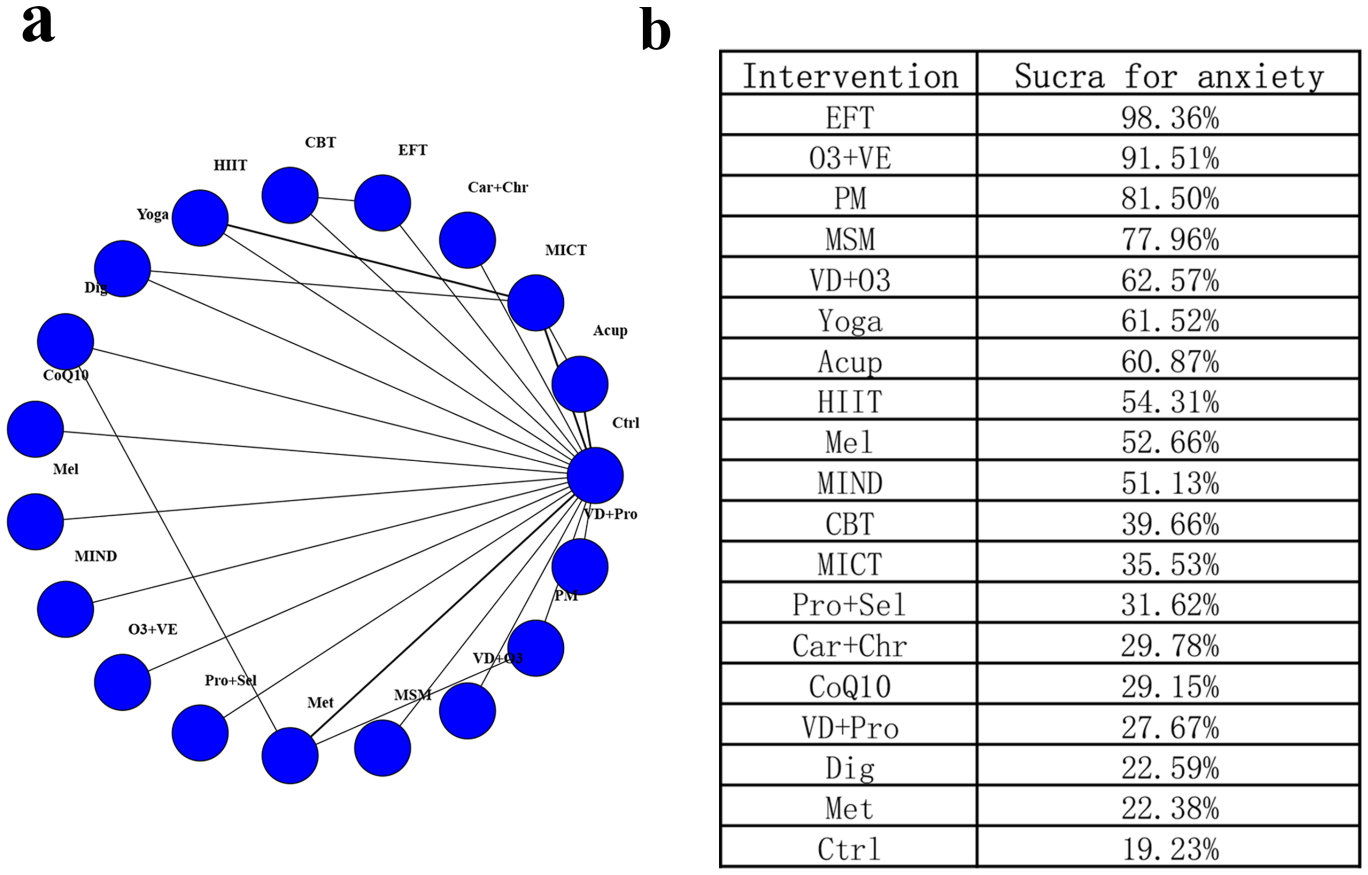
Network diagram and SUCRA values for anxiety. (A) Network diagram for anxiety; (B) SUCRA values for anxiety.

A total of 20 studies covering 1,215 participants and 19 interventions were considered for inclusion in the analysis of changes in anxiety scores (Fig. 2A). Heterogeneity across direct comparisons was low (I^2^ < 50%), and node-splitting identified no statistically significant disagreement between direct and indirect evidence (*P* > 0.05), supporting the consistency assumption (Figs. S1–S4). In comparison to the control group, EFT (SMD [95% CrI] = −2.26 [−3.33, −1.14]), O3+VE (SMD [95% CrI] = −1.55 [−2.45, −0.64]), MSM (SMD [95% CrI] = −0.99 [−1.87, −0.10]), and PM (SMD [95% CrI] = −1.07 [−1.87, −0.26]) significantly alleviated anxiety. Notably, the EFT estimate is large relative to typical psychotherapy trials and may reflect small-study effects or outcome-scale differences; therefore, it should be interpreted cautiously. For anxiety, the top-ranked interventions were EFT (SUCRA = 98.36%), O3+VE (SUCRA = 91.51%), PM (SUCRA = 81.50%), MSM (SUCRA = 77.96%), and VD+O3 (SUCRA = 62.57%). In contrast, interventions such as Met (SMD [95% CrI] = −0.02 [−0.49, 0.46]), Dig (SMD [95% CrI] = −0.01 [−0.58, 0.55]), VD+Pro (SMD [95% CrI] = −0.06 [−0.83, 0.71]), and VD+O3 (SMD [95% CrI] = −0.61 [−1.38, 0.15]) showed no statistically significant differences compared to the control. Details are presented in Fig. 2B and Fig. S5. Several nodes (*e.g.*, digital interventions) were informed by few trials in the anxiety network, and PS had no available anxiety data; therefore, estimates for these nodes should be interpreted cautiously.

### Network meta-analysis of depression

**Figure 3:**
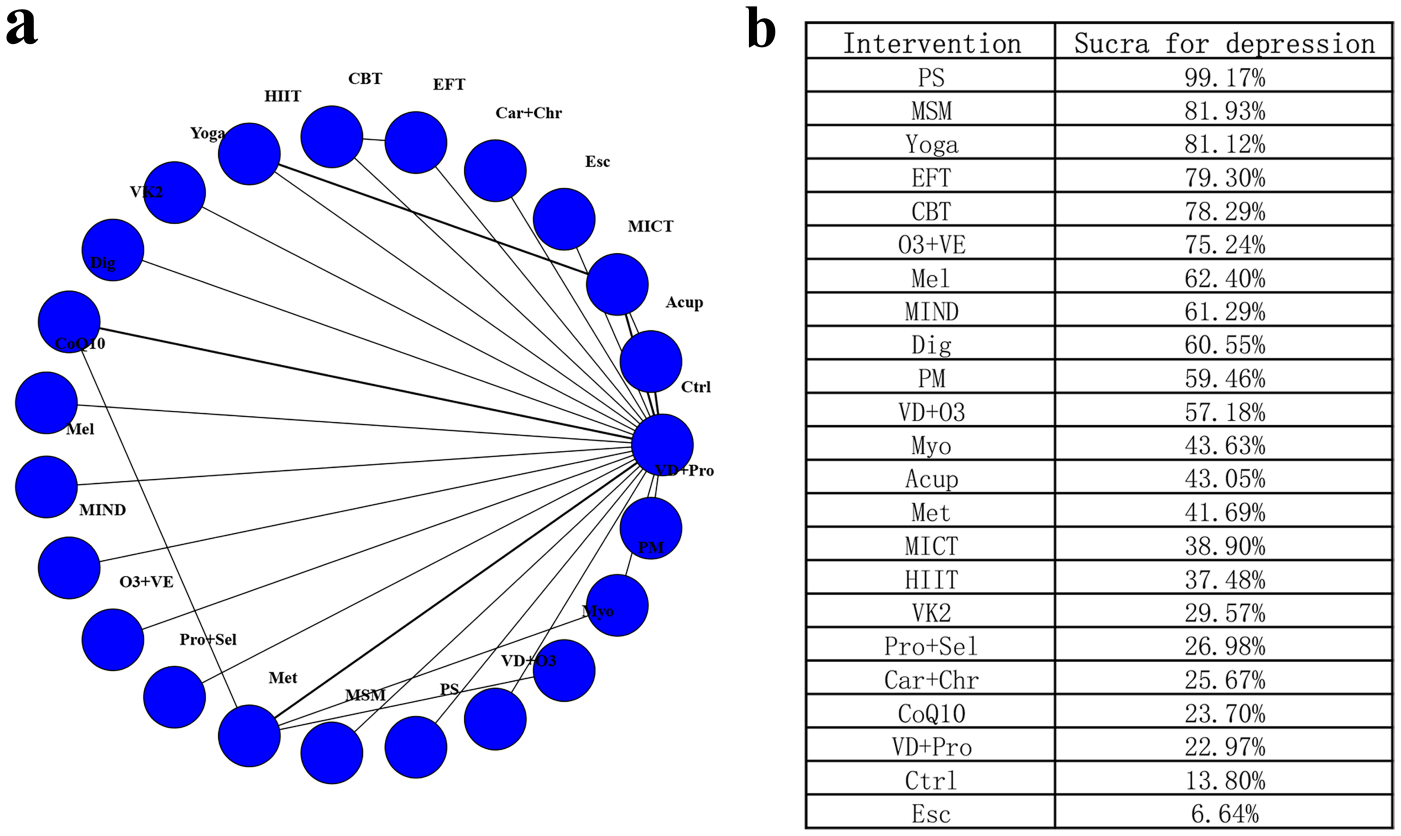
Network diagram and SUCRA values for depression. (A) Network diagram for depression; (B) SUCRA values for depression.

A total of 24 studies covering 1363 participants and 23 interventions were considered for inclusion in the analysis of changes in depression scores (Fig. 3A). Heterogeneity analysis (I^2^<50%) and node-splitting inconsistency tests (*P* > 0.05) indicated that the network meta-analysis met the hypothesis of homogeneity and consistency (Figs. S6–S9). In comparison to the control group, EFT (SMD [95% CrI] = −1.30 [−2.31, −0.28]), CBT (SMD [95% CrI] = −1.27 [−2.31, −0.23]), Yoga (SMD [95% CrI] = −1.35 [−2.32, −0.38]), Dig (SMD [95% CrI] = −0.77 [−1.29, −0.23]), O3+VE (SMD [95% CrI] = −1.16 [−2.08, −0.22]), MSM (SMD [95% CrI] = −1.37 [−2.33, −0.41]), and PS (SMD [95% CrI] = −2.71 [−3.76, −1.64]) significantly alleviated depression. For depression, the top-ranked interventions were PS (SUCRA = 99.17%), MSM (SUCRA = 81.93%), yoga (SUCRA = 81.12%), EFT (SUCRA = 79.30%), and CBT (SUCRA = 78.29%). In contrast, interventions such as CoQ10 (SMD [95% CrI] = −0.11 [−0.97, 0.72]), VD+Pro (SMD [95% CrI] = −0.11 [−0.94, 0.72]), and Esc (SMD [95% CrI] = 0.40 [−0.50, 1.29]) showed no statistically significant differences compared to the control group. Details are presented in Fig. 3B and Fig. S10. The depression estimate for PS was supported by few trials, and the effect for digital interventions likewise relied on limited studies; these findings should be considered provisional.

### SUCRA cluster analysis for anxiety and depression

**Figure 4:**
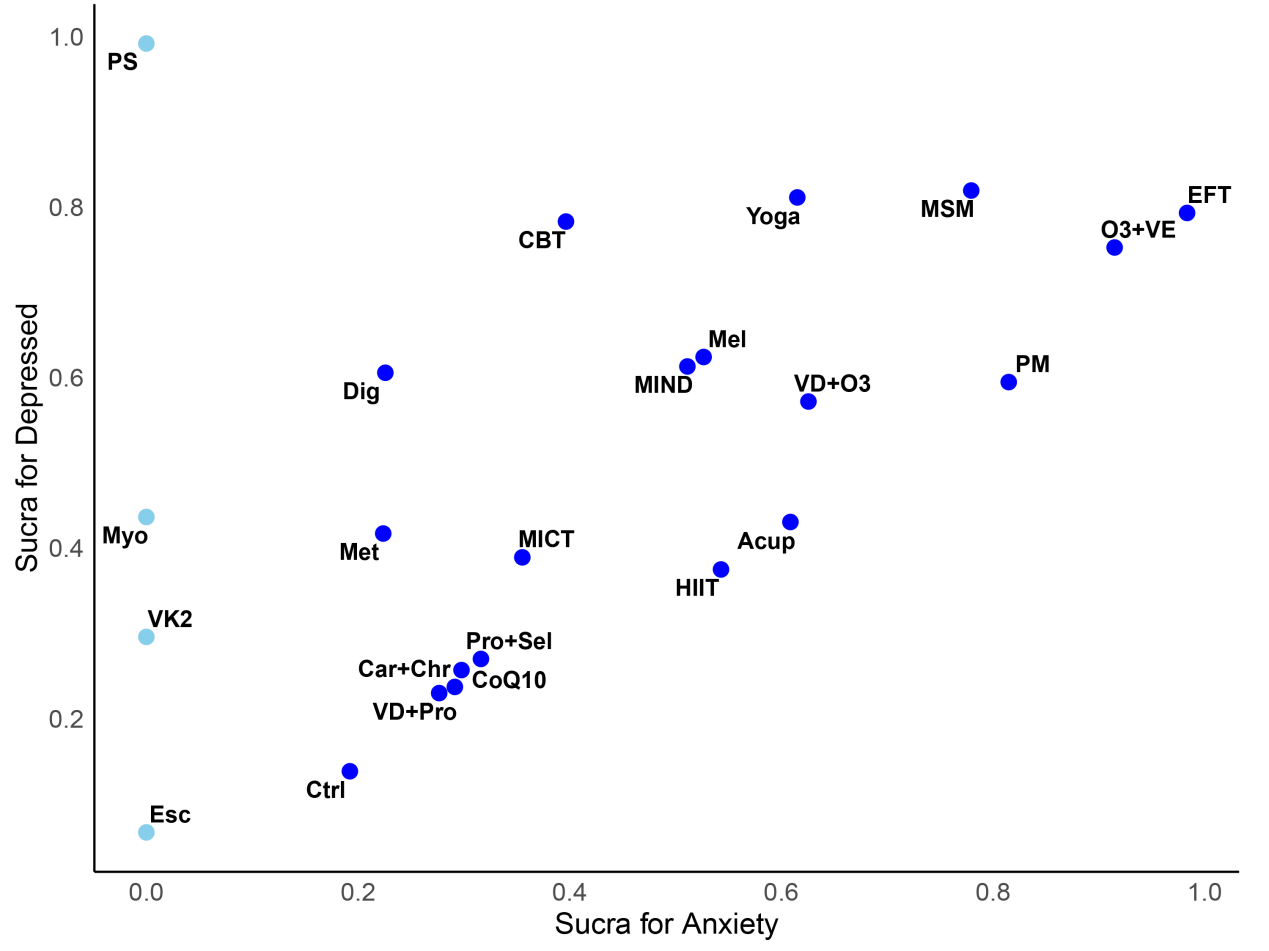
Secondary clustering diagram of SUCRA values for anxiety and depression.

The SUCRA cluster analysis plot indicated that interventions such as EFT, MSM, O3+VE, yoga, CBT, and PM showed relatively good effects on both anxiety and depression. In contrast, supplements such as VD+Pro, Car+Chr, CoQ10, and Pro+Sel demonstrated limited effectiveness. Additionally, PS appeared to have a strong effect on controlling depression; however, corresponding anxiety scores for this intervention were not available in this study. Details are presented in Fig. 4.

## Discussion

This study performed a systematic review and network meta-analysis to assess the effects of various interventions in alleviating anxiety and depression symptoms in PCOS patients. Notably, EFT, O3+VE, and MSM demonstrated prominent effects in improving both anxiety and depression. At the same time, PS demonstrated a significant effect in alleviating depression but lacked evaluation data for anxiety symptoms.

Psychological and mind–body approaches (*e.g.*, EFT, CBT, MSM, yoga) may confer larger benefits than pharmacotherapies because they directly target cognitive-affective processes, stress reactivity, and behavioral regulation relevant to PCOS-related distress, while also avoiding medication-related adverse effects that can undermine adherence. Moreover, these interventions can be individualized and integrated with lifestyle modification, potentially yielding additive or synergistic effects.

Nutrition-based and pharmacological strategies produced more heterogeneous results. Omega-3 fatty acids, particularly in combination with vitamin E, significantly reduced depressive and anxiety symptoms in women with PCOS. By contrast, other supplements such as probiotics or vitamin D formulations showed inconsistent effects, suggesting that their efficacy remains uncertain and requires further validation.

From a clinical perspective, these findings point toward the importance of personalized treatment. EFT may be prioritized in anxiety-predominant PCOS cases, while PS could be more effective in depression-predominant presentations. Mind–body interventions such as yoga or mindfulness training may be particularly beneficial for patients with mixed symptoms or those preferring non-pharmacological approaches. Personalized recommendations of this type could enhance treatment adherence and optimize outcomes. Clinicians may prioritize EFT for anxiety-predominant presentations and consider PS when depression predominates or social isolation is salient. For patients with comorbid obesity or metabolic risk, combining MSM or yoga with dietary/exercise counseling may be advantageous; cultural preferences, access to trained therapists, and willingness for group-based support should also inform selection.

It is also important to acknowledge cultural and contextual influences. For example, yoga-based therapies may be more culturally acceptable and sustainable in Southeast Asian settings (Maddock et al., 2021; Stroope et al., 2025), while structured psychotherapies such as CBT or EFT are more widely established in Western healthcare systems (Hofmann et al., 2012; Huang & Zane, 2016; Shahar, 2020). Similarly, the effectiveness of peer support may vary depending on cultural attitudes toward mental health stigma and social cohesion (Couturier et al., 2025; Treitler, Di Gioia-Laird & Long, 2025). These considerations are essential for tailoring interventions across diverse populations.

This study also has limitations. (1) the possibility of publication bias—formal assessments (*e.g.*, funnel plots) were not performed because fewer than 10 studies contributed to each direct comparison; (2) small-to-moderate sample sizes for several interventions, increasing imprecision and the potential for small-study effects; (3) heterogeneity in intervention content, intensity, and duration across trials (*e.g.*, different yoga styles or CBT structures), which may affect comparability of effect estimates and SUCRA rankings; and (4) limited evidence for some modalities (*e.g.*, digital interventions and specific supplements), warranting confirmation in larger, standardized RCTs.

Therefore, intervention regimens may be optimized and larger-scale, long-term follow-up studies may be conducted for more robust evidence in future research. Evidence for PS currently pertains only to depression, with no anxiety data available and few contributing trials; thus, generalizability is limited and confirmation in larger, adequately powered studies is needed.

## Conclusion

The results of this study indicated that EFT, O3+VE, and MSM showed significant effects in alleviating both anxiety and depression, while PS demonstrated a strong effect only in alleviating depression. CBT and yoga also showed notable efficacy for depression, ranking among the top interventions. Certain differences were observed among the interventions. Clinicians may consider EFT and mindfulness stress management as primary options for mood symptoms in PCOS, while peer support could be prioritized in depression-predominant cases; omega-3 plus vitamin E and yoga are promising complements depending on patient preferences and availability. Moreover, the focus of future studies may be investigating the underlying mechanisms of these interventions to provide more evidence-based support for psychological health interventions for these patients. Given the heterogeneity and limited number of trials for certain therapies, these findings should be interpreted with caution and considered preliminary rather than definitive.

## Supplemental Information

10.7717/peerj.20744/supp-1 Supplemental Information 1 Consistency test for anxiety Note: (1) The figures for consistency and heterogeneity tests use numbers to represent the intervention measures. The specific correspondence is as follows: 1. Control 2. Acupuncture 3. Moderate-Intensity Continuous Training 4. Escitalopram 5. Carnitine and chromium 6. Emotion-focused therapy 7. Cognitive-behavioral therapy 8. High-Intensity Interval Training 9. Yoga 10. Vitamin K2 11. Digital 12. CoQ10 13. Melatonin 14. MIND diet 15. Omega - 3 + Vitamin E 16. Probiotic + Selenium 17. Metformin 18. Mindfulness Stress Management 19. Peer Support 20. Vitamin D and Omega - 3 21. Myoinositol 22. Pioglitazone Metformin Complex 23. Vitamin D and Probiotics. The standardized mean difference (SMD) is used as the effect size. The “data.re” function, suitable for SMD analysis, was used during data analysis. The figure presented here shows the results corresponding to the SMD.

10.7717/peerj.20744/supp-2 Supplemental Information 2 Heterogeneity for Anxiety 1 Note: (1) The figures for consistency and heterogeneity tests use numbers to represent the intervention measures. The specific correspondence is as follows: 1. Control 2. Acupuncture 3. Moderate-Intensity Continuous Training 4. Escitalopram 5. Carnitine and chromium 6. Emotion-focused therapy 7. Cognitive-behavioral therapy 8. High-Intensity Interval Training 9. Yoga 10. Vitamin K2 11. Digital 12. CoQ10 13. Melatonin 14. MIND diet 15. Omega - 3 + Vitamin E 16. Probiotic + Selenium 17. Metformin 18. Mindfulness Stress Management 19. Peer Support 20. Vitamin D and Omega - 3 21. Myoinositol 22. Pioglitazone Metformin Complex 23. Vitamin D and Probiotics. The standardized mean difference (SMD) is used as the effect size. The “data.re” function, suitable for SMD analysis, was used during data analysis. The figure presented here shows the results corresponding to the SMD.

10.7717/peerj.20744/supp-3 Supplemental Information 3 Heterogeneity for Anxiety 2 Note: (1) The figures for consistency and heterogeneity tests use numbers to represent the intervention measures. The specific correspondence is as follows: 1. Control 2. Acupuncture 3. Moderate-Intensity Continuous Training 4. Escitalopram 5. Carnitine and chromium 6. Emotion-focused therapy 7. Cognitive-behavioral therapy 8. High-Intensity Interval Training 9. Yoga 10. Vitamin K2 11. Digital 12. CoQ10 13. Melatonin 14. MIND diet 15. Omega - 3 + Vitamin E 16. Probiotic + Selenium 17. Metformin 18. Mindfulness Stress Management 19. Peer Support 20. Vitamin D and Omega - 3 21. Myoinositol 22. Pioglitazone Metformin Complex 23. Vitamin D and Probiotics. The standardized mean difference (SMD) is used as the effect size. The ”data.re” function, suitable for SMD analysis, was used during data analysis. The figure presented here shows the results corresponding to the SMD.

10.7717/peerj.20744/supp-4 Supplemental Information 4 Heterogeneity for anxiety 3 Note: (1) The figures for consistency and heterogeneity tests use numbers to represent the intervention measures. The specific correspondence is as follows: 1. Control 2. Acupuncture 3. Moderate-Intensity Continuous Training 4. Escitalopram 5. Carnitine and chromium 6. Emotion-focused therapy 7. Cognitive-behavioral therapy 8. High-Intensity Interval Training 9. Yoga 10. Vitamin K2 11. Digital 12. CoQ10 13. Melatonin 14. MIND diet 15. Omega - 3 + Vitamin E 16. Probiotic + Selenium 17. Metformin 18. Mindfulness Stress Management 19. Peer Support 20. Vitamin D and Omega - 3 21. Myoinositol 22. Pioglitazone Metformin Complex 23. Vitamin D and Probiotics. The standardized mean difference (SMD) is used as the effect size. The “data.re” function, suitable for SMD analysis, was used during data analysis. The figure presented here shows the results corresponding to the SMD.

10.7717/peerj.20744/supp-5 Supplemental Information 5 League table for anxiety Note: (1) The figures for consistency and heterogeneity tests use numbers to represent the intervention measures. The specific correspondence is as follows: 1. Control 2. Acupuncture 3. Moderate-Intensity Continuous Training 4. Escitalopram 5. Carnitine and chromium 6. Emotion-focused therapy 7. Cognitive-behavioral therapy 8. High-Intensity Interval Training 9. Yoga 10. Vitamin K2 11. Digital 12. CoQ10 13. Melatonin 14. MIND diet 15. Omega - 3 + Vitamin E 16. Probiotic + Selenium 17. Metformin 18. Mindfulness Stress Management 19. Peer Support 20. Vitamin D and Omega - 3 21. Myoinositol 22. Pioglitazone Metformin Complex 23. Vitamin D and Probiotics. The standardized mean difference (SMD) is used as the effect size. The “data.re” function, suitable for SMD analysis, was used during data analysis. The figure presented here shows the results corresponding to the SMD.

10.7717/peerj.20744/supp-6 Supplemental Information 6 Consistency test for depression Note: (1) The figures for consistency and heterogeneity tests use numbers to represent the intervention measures. The specific correspondence is as follows: 1. Control 2. Acupuncture 3. Moderate-Intensity Continuous Training 4. Escitalopram 5. Carnitine and chromium 6. Emotion-focused therapy 7. Cognitive-behavioral therapy 8. High-Intensity Interval Training 9. Yoga 10. Vitamin K2 11. Digital 12. CoQ10 13. Melatonin 14. MIND diet 15. Omega - 3 + Vitamin E 16. Probiotic + Selenium 17. Metformin 18. Mindfulness Stress Management 19. Peer Support 20. Vitamin D and Omega - 3 21. Myoinositol 22. Pioglitazone Metformin Complex 23. Vitamin D and Probiotics. The standardized mean difference (SMD) is used as the effect size. The “data.re” function, suitable for SMD analysis, was used during data analysis. The figure presented here shows the results corresponding to the SMD.

10.7717/peerj.20744/supp-7 Supplemental Information 7 Heterogeneity for depression 1 Note: (1) The figures for consistency and heterogeneity tests use numbers to represent the intervention measures. The specific correspondence is as follows: 1.Control 2. Acupuncture 3. Moderate-Intensity Continuous Training 4. Escitalopram 5. Carnitine and chromium 6. Emotion-focused therapy 7. Cognitive-behavioral therapy 8. High-Intensity Interval Training 9. Yoga 10. Vitamin K2 11. Digital 12. CoQ10 13. Melatonin 14. MIND diet 15. Omega - 3 + Vitamin E 16. Probiotic + Selenium 17. Metformin 18. Mindfulness Stress Management 19. Peer Support 20. Vitamin D and Omega - 3 21. Myoinositol 22. Pioglitazone Metformin Complex 23. Vitamin D and Probiotics. The standardized mean difference (SMD) is used as the effect size. The “data.re” function, suitable for SMD analysis, was used during data analysis. The figure presented here shows the results corresponding to the SMD.

10.7717/peerj.20744/supp-8 Supplemental Information 8 Heterogeneity for depression 2 Note: (1) The figures for consistency and heterogeneity tests use numbers to represent the intervention measures. The specific correspondence is as follows: 1.Control 2. Acupuncture 3. Moderate-Intensity Continuous Training 4. Escitalopram 5. Carnitine and chromium 6. Emotion-focused therapy 7. Cognitive-behavioral therapy 8. High-Intensity Interval Training 9. Yoga 10. Vitamin K2 11. Digital 12. CoQ10 13. Melatonin 14. MIND diet 15. Omega - 3 + Vitamin E 16. Probiotic + Selenium 17. Metformin 18. Mindfulness Stress Management 19. Peer Support 20. Vitamin D and Omega - 3 21. Myoinositol 22. Pioglitazone Metformin Complex 23. Vitamin D and Probiotics. The standardized mean difference (SMD) is used as the effect size. The “data.re” function, suitable for SMD analysis, was used during data analysis. The figure presented here shows the results corresponding to the SMD.

10.7717/peerj.20744/supp-9 Supplemental Information 9 Heterogeneity for depression 3 Note: (1) The figures for consistency and heterogeneity tests use numbers to represent the intervention measures. The specific correspondence is as follows: 1.Control 2. Acupuncture 3. Moderate-Intensity Continuous Training 4. Escitalopram 5. Carnitine and chromium 6. Emotion-focused therapy 7. Cognitive-behavioral therapy 8. High-Intensity Interval Training 9. Yoga 10. Vitamin K2 11. Digital 12. CoQ10 13. Melatonin 14. MIND diet 15. Omega - 3 + Vitamin E 16. Probiotic + Selenium 17. Metformin 18. Mindfulness Stress Management 19. Peer Support 20. Vitamin D and Omega - 3 21. Myoinositol 22. Pioglitazone Metformin Complex 23. Vitamin D and Probiotics. The standardized mean difference (SMD) is used as the effect size. The “data.re” function, suitable for SMD analysis, was used during data analysis. The figure presented here shows the results corresponding to the SMD.

10.7717/peerj.20744/supp-10 Supplemental Information 10 League table for depression Note: (1) The figures for consistency and heterogeneity tests use numbers to represent the intervention measures. The specific correspondence is as follows: 1.Control 2. Acupuncture 3. Moderate-Intensity Continuous Training 4. Escitalopram 5. Carnitine and chromium 6. Emotion-focused therapy 7. Cognitive-behavioral therapy 8. High-Intensity Interval Training 9. Yoga 10. Vitamin K2 11. Digital 12. CoQ10 13. Melatonin 14. MIND diet 15. Omega - 3 + Vitamin E 16. Probiotic + Selenium 17. Metformin 18. Mindfulness Stress Management 19. Peer Support 20. Vitamin D and Omega - 3 21. Myoinositol 22. Pioglitazone Metformin Complex 23. Vitamin D and Probiotics. The standardized mean difference (SMD) is used as the effect size. The “data.re” function, suitable for SMD analysis, was used during data analysis. The figure presented here shows the results corresponding to the SMD.

10.7717/peerj.20744/supp-11 Supplemental Information 11 PRISMA checklist

10.7717/peerj.20744/supp-12 Supplemental Information 12 Search strategy for network meta-analysis

10.7717/peerj.20744/supp-13 Supplemental Information 13 Excluded Studies and Reasons After Reviewing the Full Text

10.7717/peerj.20744/supp-14 Supplemental Information 14 Systematic Review and/or Meta-Analysis Rationale

10.7717/peerj.20744/supp-15 Supplemental Information 15 Graphical Abstract Summarizes the study selection process and the relative efficacy of different interventions for anxiety and depression based on SUCRA rankings from the network meta-analysis.

## Additional Information and Declarations

### Data Availability

The following information was supplied regarding data availability: This is a systematic review/meta-analysis.
