# Supplementary figures and images for: A network meta-analysis of interventions for anxiety and depression in PCOS
Source: PeerJ. 2026 Feb 5;14:e20744. doi: 10.7717/peerj.20744 (PMC12883158; doi:10.7717/peerj.20744)

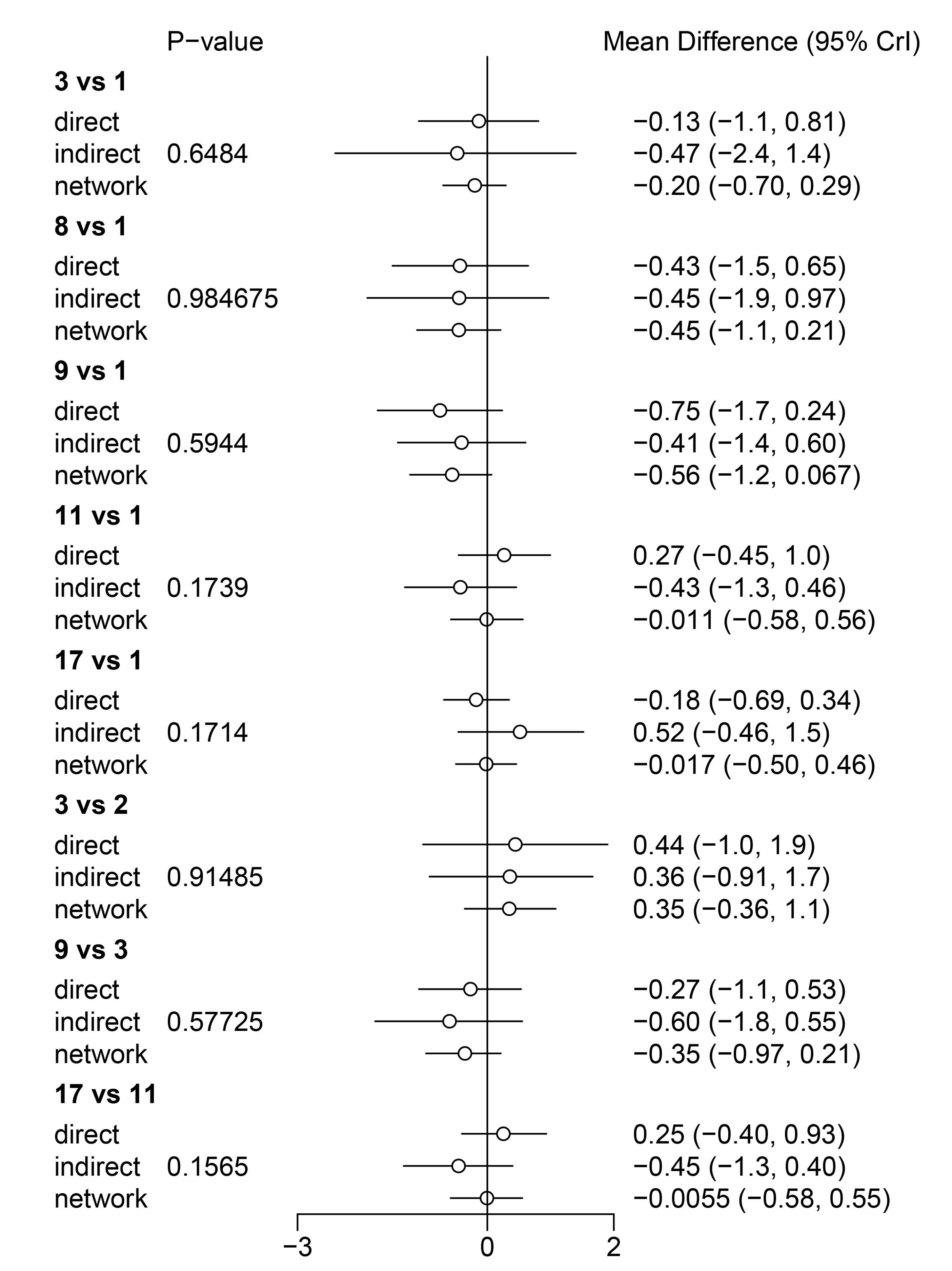

Supplement: Supplemental Information 1 — Note: (1) The figures for consistency and heterogeneity tests use numbers to represent the intervention measures. The specific correspondence is as follows: 1. Control 2. Acupuncture 3. Moderate-Intensity Continuous Training 4. Escitalopram 5. Carnitine and chromium 6. Emotion-focused therapy 7. Cognitive-behavioral therapy 8. High-Intensity Interval Training 9. Yoga 10. Vitamin K2 11. Digital 12. CoQ10 13. Melatonin 14. MIND diet 15. Omega - 3 + Vitamin E 16. Probiotic + Selenium 17. Metformin 18. Mindfulness Stress Management 19. Peer Support 20. Vitamin D and Omega - 3 21. Myoinositol 22. Pioglitazone Metformin Complex 23. Vitamin D and Probiotics. The standardized mean difference (SMD) is used as the effect size. The “data.re” function, suitable for SMD analysis, was used during data analysis. The figure presented here shows the results corresponding to the SMD. [file peerj-14-20744-s001.png]

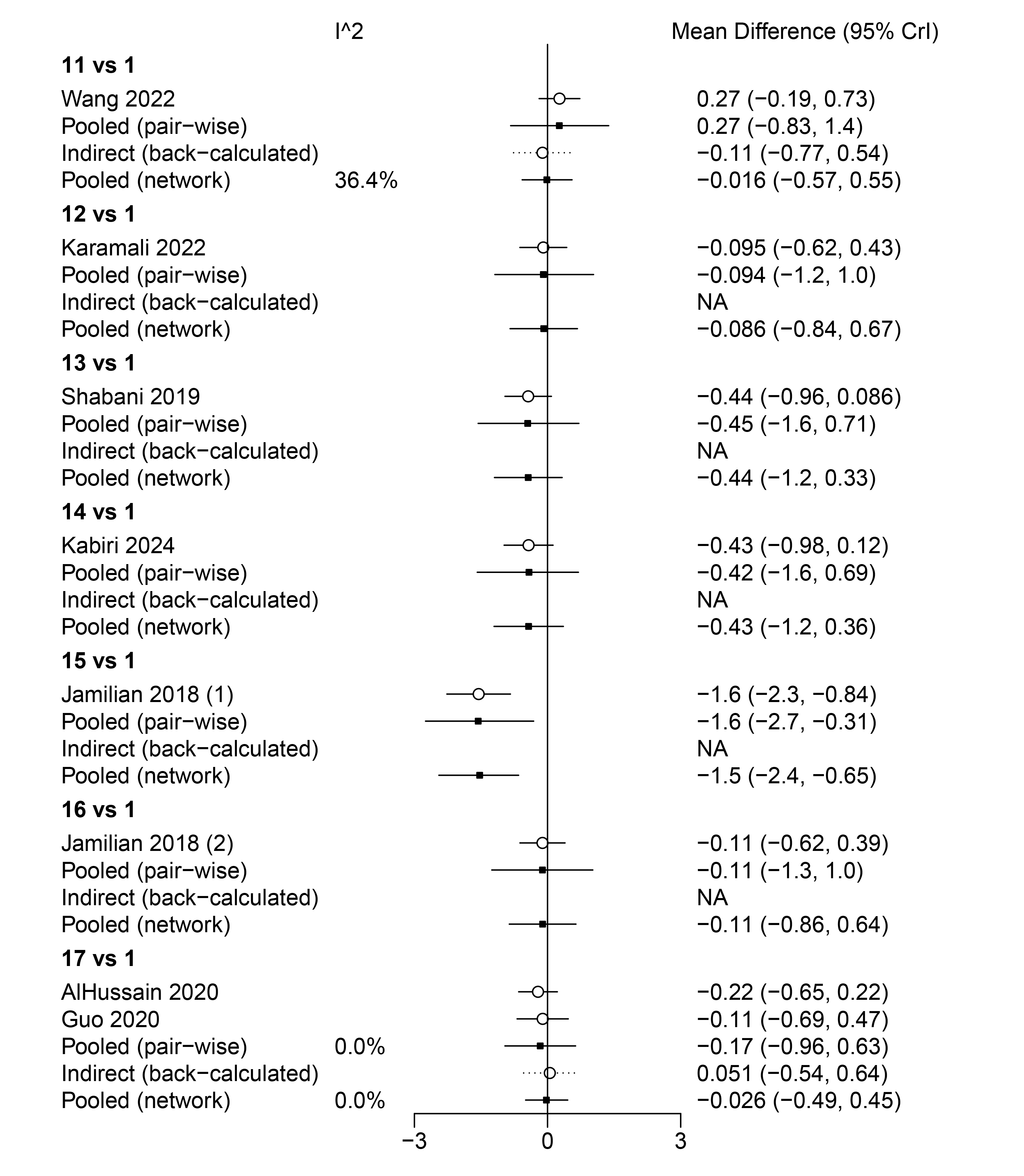

Supplement: Supplemental Information 2 — Note: (1) The figures for consistency and heterogeneity tests use numbers to represent the intervention measures. The specific correspondence is as follows: 1. Control 2. Acupuncture 3. Moderate-Intensity Continuous Training 4. Escitalopram 5. Carnitine and chromium 6. Emotion-focused therapy 7. Cognitive-behavioral therapy 8. High-Intensity Interval Training 9. Yoga 10. Vitamin K2 11. Digital 12. CoQ10 13. Melatonin 14. MIND diet 15. Omega - 3 + Vitamin E 16. Probiotic + Selenium 17. Metformin 18. Mindfulness Stress Management 19. Peer Support 20. Vitamin D and Omega - 3 21. Myoinositol 22. Pioglitazone Metformin Complex 23. Vitamin D and Probiotics. The standardized mean difference (SMD) is used as the effect size. The “data.re” function, suitable for SMD analysis, was used during data analysis. The figure presented here shows the results corresponding to the SMD. [file peerj-14-20744-s002.png]

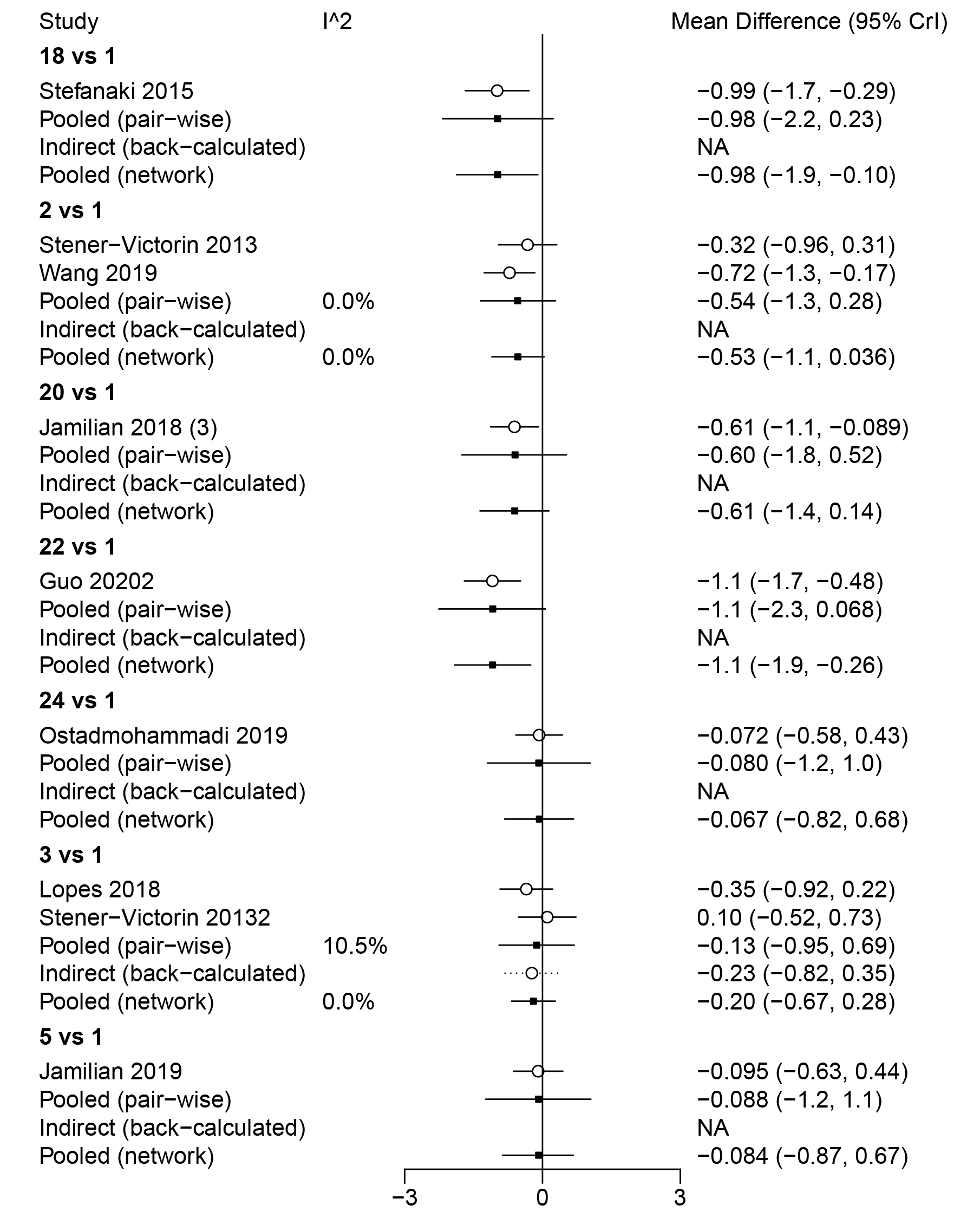

Supplement: Supplemental Information 3 — Note: (1) The figures for consistency and heterogeneity tests use numbers to represent the intervention measures. The specific correspondence is as follows: 1. Control 2. Acupuncture 3. Moderate-Intensity Continuous Training 4. Escitalopram 5. Carnitine and chromium 6. Emotion-focused therapy 7. Cognitive-behavioral therapy 8. High-Intensity Interval Training 9. Yoga 10. Vitamin K2 11. Digital 12. CoQ10 13. Melatonin 14. MIND diet 15. Omega - 3 + Vitamin E 16. Probiotic + Selenium 17. Metformin 18. Mindfulness Stress Management 19. Peer Support 20. Vitamin D and Omega - 3 21. Myoinositol 22. Pioglitazone Metformin Complex 23. Vitamin D and Probiotics. The standardized mean difference (SMD) is used as the effect size. The ”data.re” function, suitable for SMD analysis, was used during data analysis. The figure presented here shows the results corresponding to the SMD. [file peerj-14-20744-s003.png]

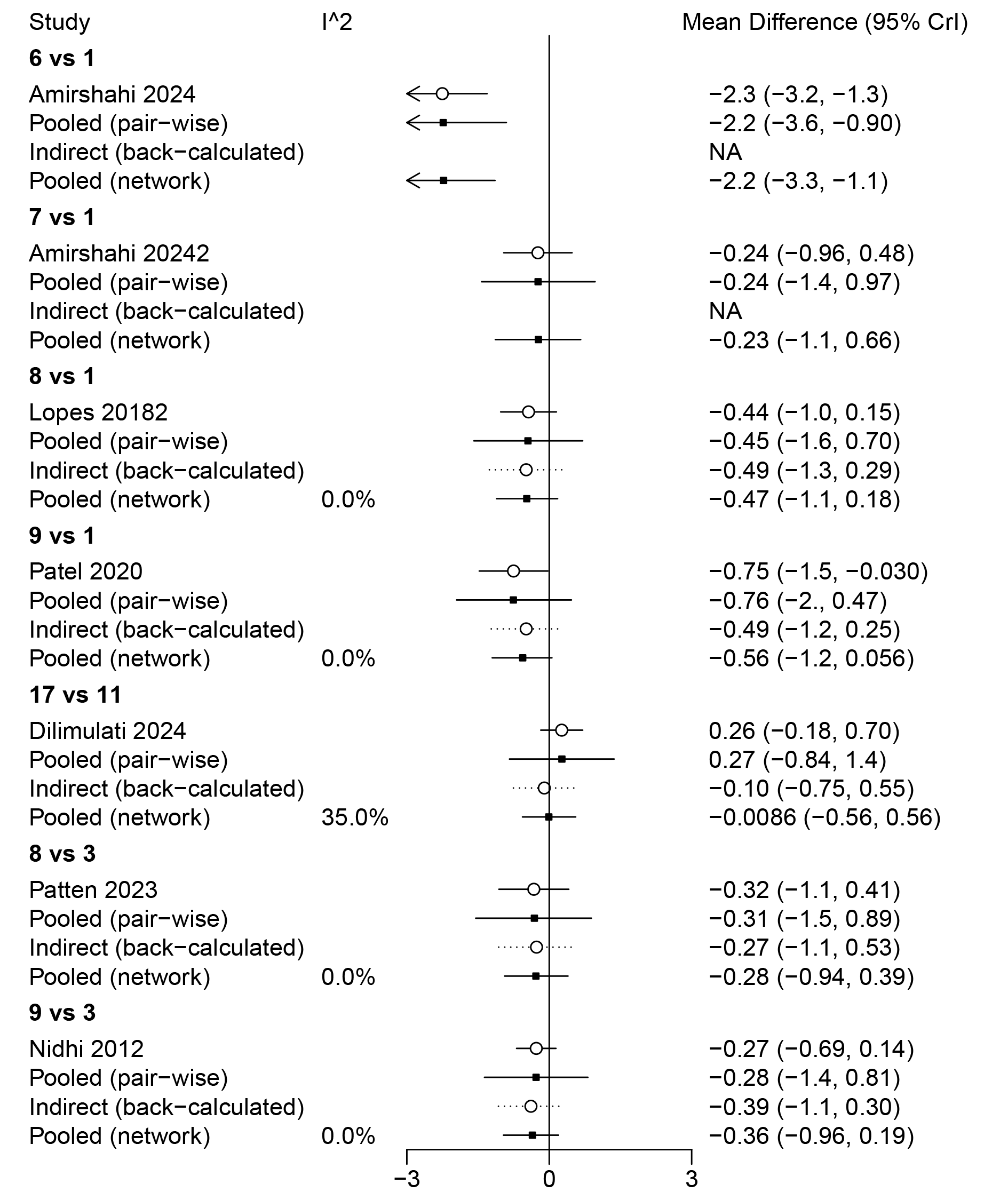

Supplement: Supplemental Information 4 — Note: (1) The figures for consistency and heterogeneity tests use numbers to represent the intervention measures. The specific correspondence is as follows: 1. Control 2. Acupuncture 3. Moderate-Intensity Continuous Training 4. Escitalopram 5. Carnitine and chromium 6. Emotion-focused therapy 7. Cognitive-behavioral therapy 8. High-Intensity Interval Training 9. Yoga 10. Vitamin K2 11. Digital 12. CoQ10 13. Melatonin 14. MIND diet 15. Omega - 3 + Vitamin E 16. Probiotic + Selenium 17. Metformin 18. Mindfulness Stress Management 19. Peer Support 20. Vitamin D and Omega - 3 21. Myoinositol 22. Pioglitazone Metformin Complex 23. Vitamin D and Probiotics. The standardized mean difference (SMD) is used as the effect size. The “data.re” function, suitable for SMD analysis, was used during data analysis. The figure presented here shows the results corresponding to the SMD. [file peerj-14-20744-s004.png]

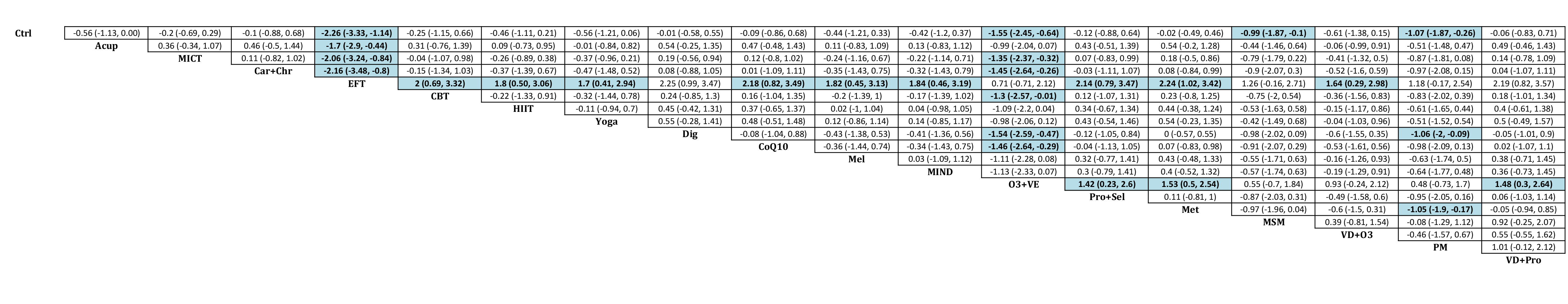

Supplement: Supplemental Information 5 — Note: (1) The figures for consistency and heterogeneity tests use numbers to represent the intervention measures. The specific correspondence is as follows: 1. Control 2. Acupuncture 3. Moderate-Intensity Continuous Training 4. Escitalopram 5. Carnitine and chromium 6. Emotion-focused therapy 7. Cognitive-behavioral therapy 8. High-Intensity Interval Training 9. Yoga 10. Vitamin K2 11. Digital 12. CoQ10 13. Melatonin 14. MIND diet 15. Omega - 3 + Vitamin E 16. Probiotic + Selenium 17. Metformin 18. Mindfulness Stress Management 19. Peer Support 20. Vitamin D and Omega - 3 21. Myoinositol 22. Pioglitazone Metformin Complex 23. Vitamin D and Probiotics. The standardized mean difference (SMD) is used as the effect size. The “data.re” function, suitable for SMD analysis, was used during data analysis. The figure presented here shows the results corresponding to the SMD. [file peerj-14-20744-s005.png]

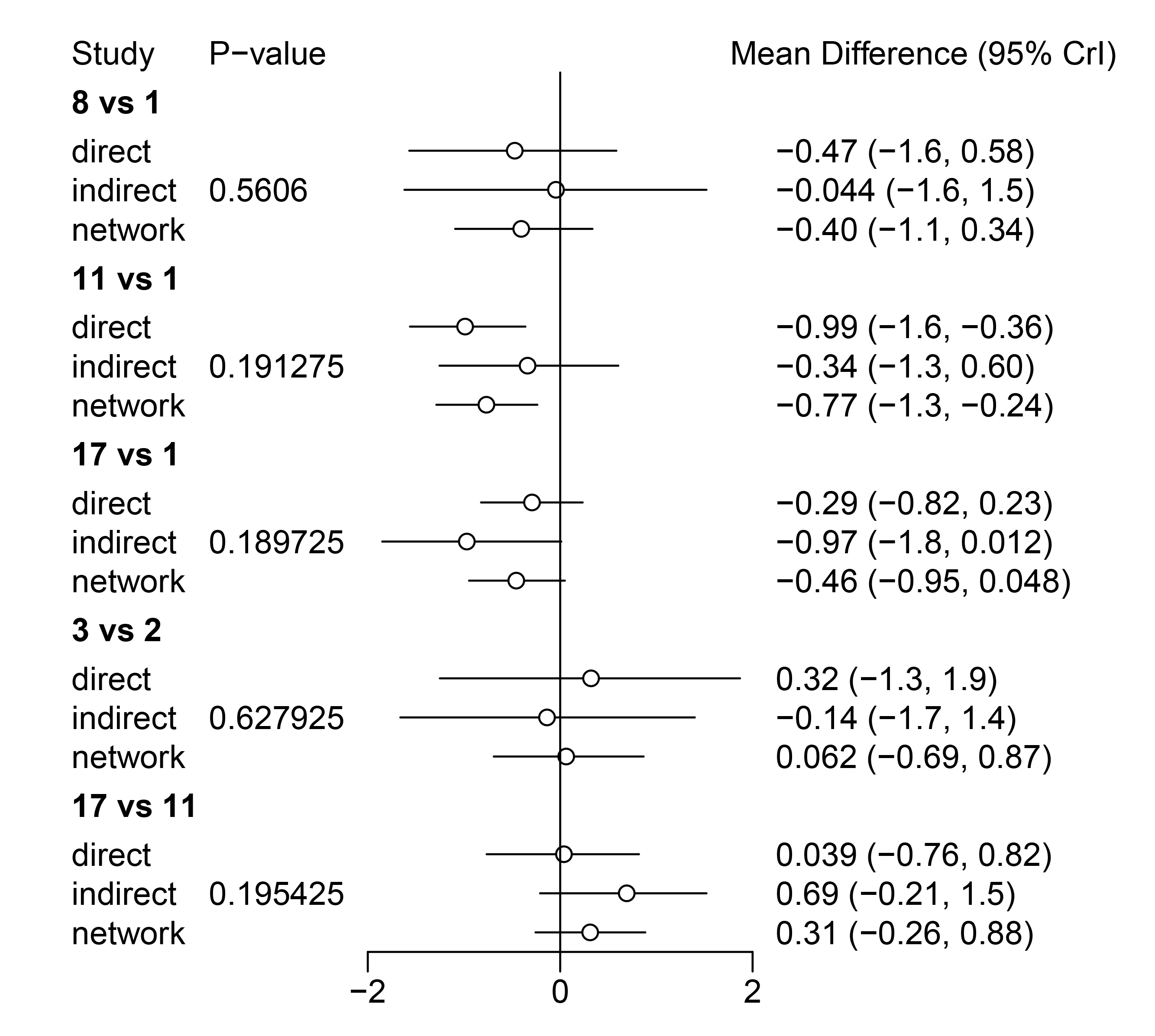

Supplement: Supplemental Information 6 — Note: (1) The figures for consistency and heterogeneity tests use numbers to represent the intervention measures. The specific correspondence is as follows: 1. Control 2. Acupuncture 3. Moderate-Intensity Continuous Training 4. Escitalopram 5. Carnitine and chromium 6. Emotion-focused therapy 7. Cognitive-behavioral therapy 8. High-Intensity Interval Training 9. Yoga 10. Vitamin K2 11. Digital 12. CoQ10 13. Melatonin 14. MIND diet 15. Omega - 3 + Vitamin E 16. Probiotic + Selenium 17. Metformin 18. Mindfulness Stress Management 19. Peer Support 20. Vitamin D and Omega - 3 21. Myoinositol 22. Pioglitazone Metformin Complex 23. Vitamin D and Probiotics. The standardized mean difference (SMD) is used as the effect size. The “data.re” function, suitable for SMD analysis, was used during data analysis. The figure presented here shows the results corresponding to the SMD. [file peerj-14-20744-s006.png]

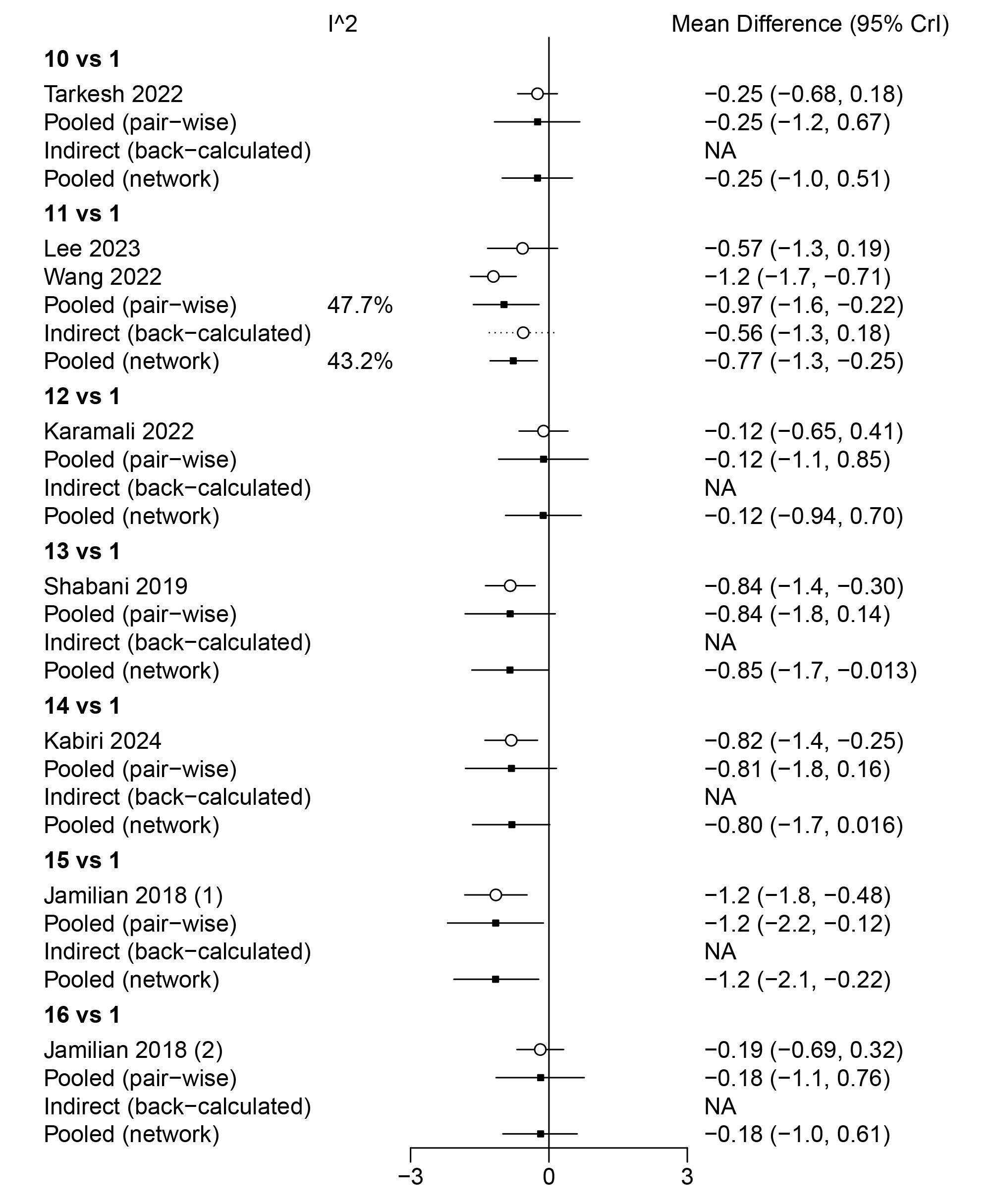

Supplement: Supplemental Information 7 — Note: (1) The figures for consistency and heterogeneity tests use numbers to represent the intervention measures. The specific correspondence is as follows: 1.Control 2. Acupuncture 3. Moderate-Intensity Continuous Training 4. Escitalopram 5. Carnitine and chromium 6. Emotion-focused therapy 7. Cognitive-behavioral therapy 8. High-Intensity Interval Training 9. Yoga 10. Vitamin K2 11. Digital 12. CoQ10 13. Melatonin 14. MIND diet 15. Omega - 3 + Vitamin E 16. Probiotic + Selenium 17. Metformin 18. Mindfulness Stress Management 19. Peer Support 20. Vitamin D and Omega - 3 21. Myoinositol 22. Pioglitazone Metformin Complex 23. Vitamin D and Probiotics. The standardized mean difference (SMD) is used as the effect size. The “data.re” function, suitable for SMD analysis, was used during data analysis. The figure presented here shows the results corresponding to the SMD. [file peerj-14-20744-s007.png]

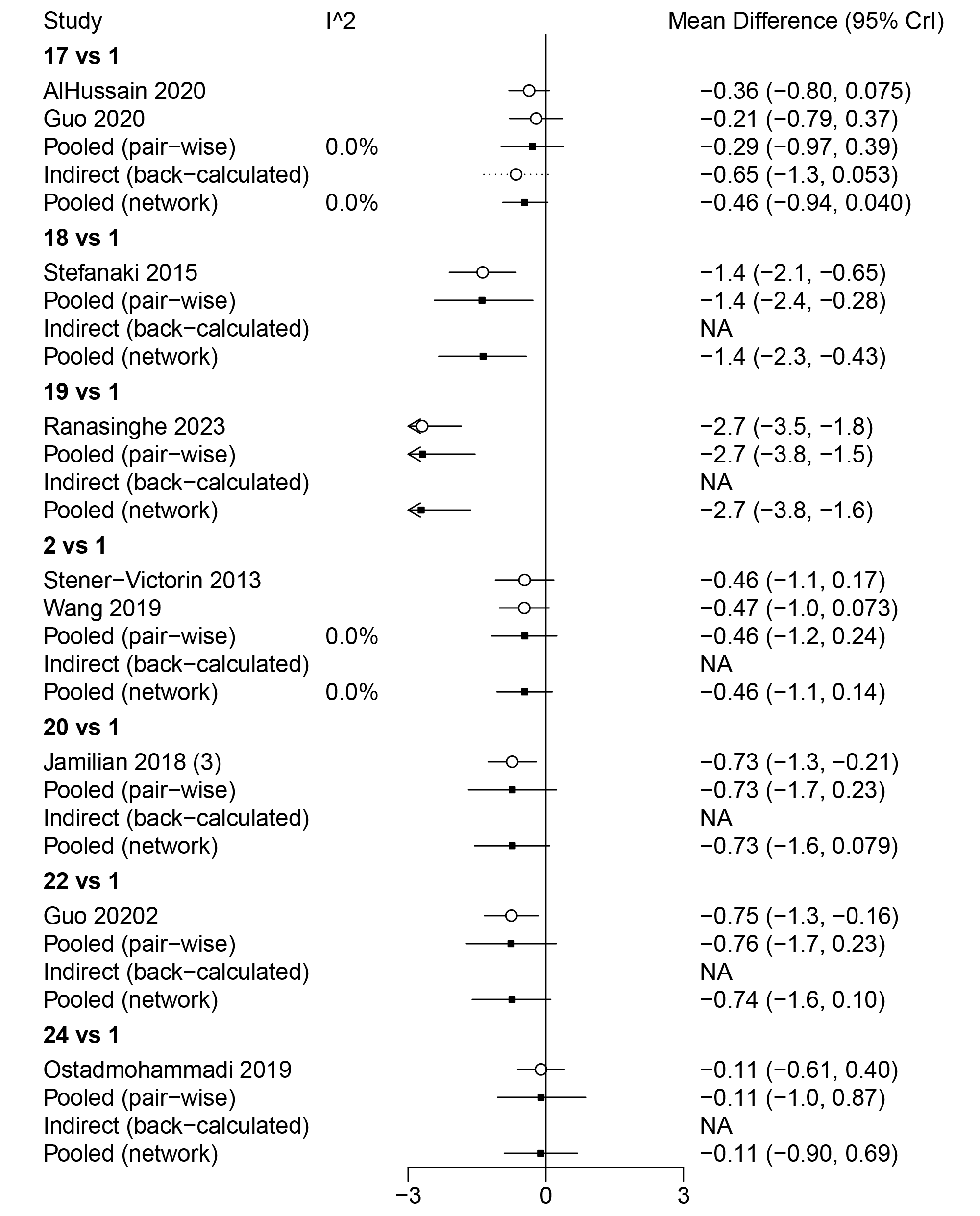

Supplement: Supplemental Information 8 — Note: (1) The figures for consistency and heterogeneity tests use numbers to represent the intervention measures. The specific correspondence is as follows: 1.Control 2. Acupuncture 3. Moderate-Intensity Continuous Training 4. Escitalopram 5. Carnitine and chromium 6. Emotion-focused therapy 7. Cognitive-behavioral therapy 8. High-Intensity Interval Training 9. Yoga 10. Vitamin K2 11. Digital 12. CoQ10 13. Melatonin 14. MIND diet 15. Omega - 3 + Vitamin E 16. Probiotic + Selenium 17. Metformin 18. Mindfulness Stress Management 19. Peer Support 20. Vitamin D and Omega - 3 21. Myoinositol 22. Pioglitazone Metformin Complex 23. Vitamin D and Probiotics. The standardized mean difference (SMD) is used as the effect size. The “data.re” function, suitable for SMD analysis, was used during data analysis. The figure presented here shows the results corresponding to the SMD. [file peerj-14-20744-s008.png]

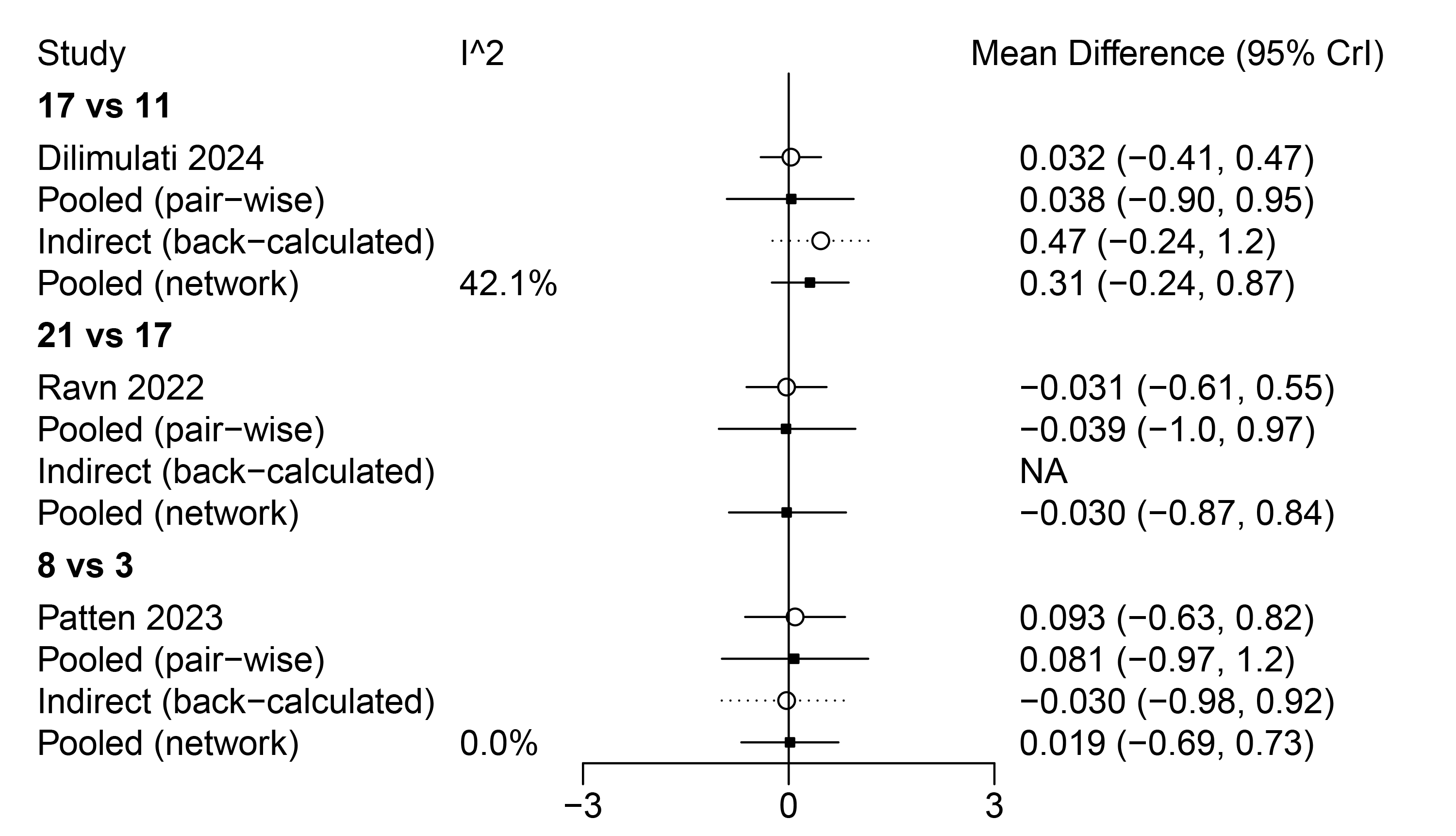

Supplement: Supplemental Information 9 — Note: (1) The figures for consistency and heterogeneity tests use numbers to represent the intervention measures. The specific correspondence is as follows: 1.Control 2. Acupuncture 3. Moderate-Intensity Continuous Training 4. Escitalopram 5. Carnitine and chromium 6. Emotion-focused therapy 7. Cognitive-behavioral therapy 8. High-Intensity Interval Training 9. Yoga 10. Vitamin K2 11. Digital 12. CoQ10 13. Melatonin 14. MIND diet 15. Omega - 3 + Vitamin E 16. Probiotic + Selenium 17. Metformin 18. Mindfulness Stress Management 19. Peer Support 20. Vitamin D and Omega - 3 21. Myoinositol 22. Pioglitazone Metformin Complex 23. Vitamin D and Probiotics. The standardized mean difference (SMD) is used as the effect size. The “data.re” function, suitable for SMD analysis, was used during data analysis. The figure presented here shows the results corresponding to the SMD. [file peerj-14-20744-s009.png]

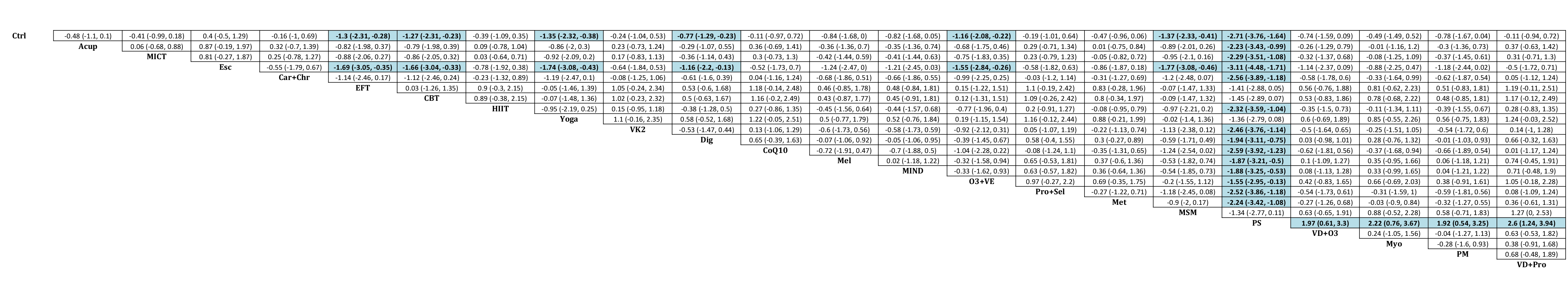

Supplement: Supplemental Information 10 — Note: (1) The figures for consistency and heterogeneity tests use numbers to represent the intervention measures. The specific correspondence is as follows: 1.Control 2. Acupuncture 3. Moderate-Intensity Continuous Training 4. Escitalopram 5. Carnitine and chromium 6. Emotion-focused therapy 7. Cognitive-behavioral therapy 8. High-Intensity Interval Training 9. Yoga 10. Vitamin K2 11. Digital 12. CoQ10 13. Melatonin 14. MIND diet 15. Omega - 3 + Vitamin E 16. Probiotic + Selenium 17. Metformin 18. Mindfulness Stress Management 19. Peer Support 20. Vitamin D and Omega - 3 21. Myoinositol 22. Pioglitazone Metformin Complex 23. Vitamin D and Probiotics. The standardized mean difference (SMD) is used as the effect size. The “data.re” function, suitable for SMD analysis, was used during data analysis. The figure presented here shows the results corresponding to the SMD. [file peerj-14-20744-s010.png]

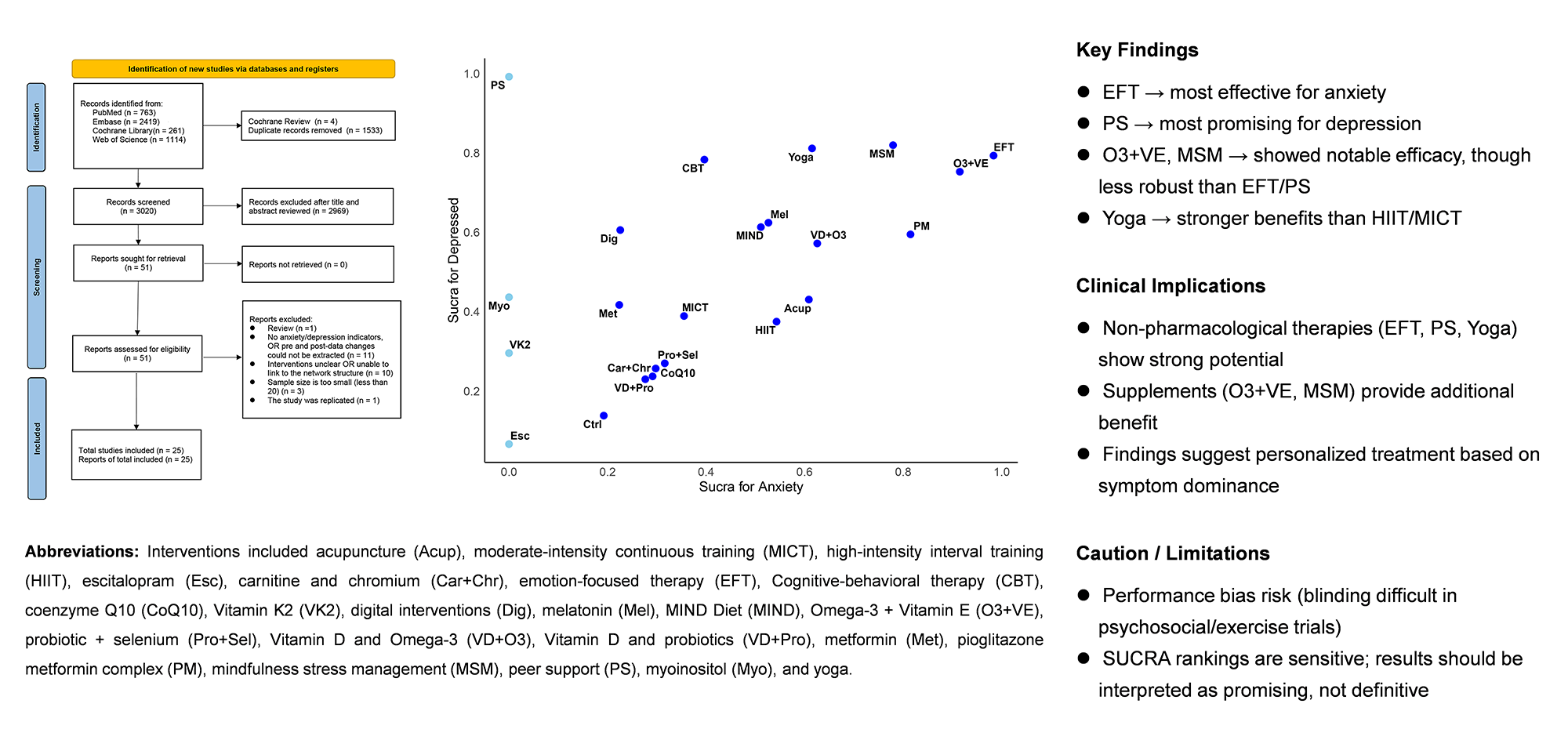

Supplement: Supplemental Information 15 — Summarizes the study selection process and the relative efficacy of different interventions for anxiety and depression based on SUCRA rankings from the network meta-analysis. [file peerj-14-20744-s015.png]
