# Supplementary material for: A network meta-analysis of interventions for anxiety and depression in PCOS
Source: PeerJ. 2026 Feb 5;14:e20744. doi: 10.7717/peerj.20744 (PMC12883158; doi:10.7717/peerj.20744)
Supplement: Supplemental Information 12 [file peerj-14-20744-s012.docx]

**Table S1:**

**Search strategy for network meta-analysis.**

**1.1 Search strategy of Pubmed.**

| Pubmed | | |
| --- | --- | --- |
| # | Query | Results |
| #1 | "polycystic ovary syndrome"[MeSH Terms] | 19516 |
| #2 | "syndrome stein leventhal"[Title/Abstract] OR "Stein Leventhal syndrome"[Title/Abstract] OR "Sclerocystic Ovary Syndrome"[Title/Abstract] OR "Sclerocystic Ovary"[Title/Abstract] OR "Sclerocystic Ovaries"[Title/Abstract] OR "Sclerocystic Ovarian Degeneration"[Title/Abstract] OR "polycystic ovary syndrome"[Title/Abstract] OR "polycystic ovary disease"[Title/Abstract] OR "polycystic ovary"[Title/Abstract] OR "Polycystic Ovarian Syndrome"[Title/Abstract] OR "polycystic ovarian disease"[Title/Abstract] OR "ovary polycystic disease"[Title/Abstract] OR "micropolycystic ovary"[Title/Abstract] OR "cystic ovary"[Title/Abstract] | 23960 |
| #3 | "depressive disorder"[MeSH Terms] OR "depression"[MeSH Terms] | 274383 |
| #4 | "anxiety"[MeSH Terms] | 123208 |
| #5 | "parental depression"[Title/Abstract] OR "mental depression"[Title/Abstract] OR "Emotional Depression"[Title/Abstract] OR "depressivity"[Title/Abstract] OR "depressive syndrome"[Title/Abstract] OR "Depressive Symptoms"[Title/Abstract] OR "depressive symptom"[Title/Abstract] OR "depressive state"[Title/Abstract] OR "depressive personality disorder"[Title/Abstract] OR "depressive illness"[Title/Abstract] OR "depressive episode"[Title/Abstract] OR "depressive disorder"[Title/Abstract] OR "depressive disease"[Title/Abstract] OR "depression"[Title/Abstract] OR "clinical depression"[Title/Abstract] OR "central depression"[Title/Abstract] OR "Social Anxiety"[Title/Abstract] OR "Social Anxieties"[Title/Abstract] OR "Nervousness"[Title/Abstract] OR "Hypervigilance"[Title/Abstract] OR "Anxiousness"[Title/Abstract] OR "anxiety"[Title/Abstract] OR "Angst"[Title/Abstract] | 661366 |
| #6 | (#1 OR #2) AND (#3 OR #4 OR #5) | 763 |

**1.2 Search strategy of Embase.**

| Embase | | |
| --- | --- | --- |
| # | Query | Results |
| #1 | 'ovary polycystic disease'/exp | 41703 |
| #2 | 'syndrome stein leventhal':ti,ab,kw OR 'stein leventhal syndrome':ti,ab,kw OR 'stein leventhal disease':ti,ab,kw OR 'stein cohen leventhal syndrome':ti,ab,kw OR 'sclerocystic ovary syndrome':ti,ab,kw OR 'sclerocystic ovary':ti,ab,kw OR 'sclerocystic ovaries':ti,ab,kw OR 'sclerocystic ovarian degeneration':ti,ab,kw OR 'polycystic ovary syndrome':ti,ab,kw OR 'polycystic ovary disease':ti,ab,kw OR 'polycystic ovary':ti,ab,kw OR 'polycystic ovarian syndrome':ti,ab,kw OR 'polycystic ovarian disease':ti,ab,kw OR 'ovary polycystic syndrome':ti,ab,kw OR 'ovary polycystic disease':ti,ab,kw OR 'multiple follicle cyst':ti,ab,kw OR 'micropolycystic ovary':ti,ab,kw OR 'cystic ovary':ti,ab,kw | 34087 |
| #3 | 'depression'/exp | 715362 |
| #4 | 'anxiety'/exp | 338955 |
| #5 | 'parental depression':ti,ab,kw OR 'mental depression':ti,ab,kw OR 'emotional depression':ti,ab,kw OR 'depressivity':ti,ab,kw OR 'depressive syndrome':ti,ab,kw OR 'depressive symptoms':ti,ab,kw OR 'depressive symptom':ti,ab,kw OR 'depressive state':ti,ab,kw OR 'depressive personality disorder':ti,ab,kw OR 'depressive illness':ti,ab,kw OR 'depressive episode':ti,ab,kw OR 'depressive disorder':ti,ab,kw OR 'depressive disease':ti,ab,kw OR 'depression':ti,ab,kw OR 'clinical depression':ti,ab,kw OR 'central depression':ti,ab,kw OR 'social anxiety':ti,ab,kw OR 'social anxieties':ti,ab,kw OR 'nervousness':ti,ab,kw OR 'hypervigilance':ti,ab,kw OR 'anxiousness':ti,ab,kw OR 'anxiety':ti,ab,kw OR 'angst':ti,ab,kw | 908426 |
| #6 | (#1 OR #2) AND (#3 OR #4 OR #5) | 2419 |

**1.3 Search strategy of Cochrane Library.**

| Cochrane Library | | |
| --- | --- | --- |
| # | Query | Results |
| #1 | MeSH descriptor: [Polycystic Ovary Syndrome] explode all trees | 2207 |
| #2 | ('syndrome stein leventhal' OR 'Stein Leventhal syndrome' OR 'stein leventhal disease' OR 'stein cohen leventhal syndrome' OR 'Sclerocystic Ovary Syndrome' OR 'Sclerocystic Ovary' OR 'Sclerocystic Ovaries' OR 'Sclerocystic Ovarian Degeneration' OR 'polycystic ovary syndrome' OR 'polycystic ovary disease' OR 'polycystic ovary' OR 'Polycystic Ovarian Syndrome' OR 'polycystic ovarian disease' OR 'ovary polycystic syndrome' OR 'ovary polycystic disease' OR 'multiple follicle cyst' OR 'micropolycystic ovary' OR 'cystic ovary'):ti,ab,kw | 5490 |
| #3 | MeSH descriptor: [Depression] explode all trees | 19070 |
| #4 | MeSH descriptor: [Anxiety] explode all trees | 13320 |
| #5 | ('parental depression' OR 'mental depression' OR 'Emotional Depression' OR 'depressivity' OR 'depressive syndrome' OR 'Depressive Symptoms' OR 'depressive symptom' OR 'depressive state' OR 'depressive personality disorder' OR 'depressive illness' OR 'depressive episode' OR 'depressive disorder' OR 'depressive disease' OR 'depression' OR 'clinical depression' OR 'central depression' OR 'Social Anxiety' OR 'Social Anxieties' OR 'Nervousness' OR 'Hypervigilance' OR 'Anxiousness' OR 'anxiety' OR 'Angst'):ti,ab,kw | 155895 |
| #6 | (#1 OR #2) AND (#3 OR #4 OR #5) | 261 |

**1.4 Search strategy of Web of Science.**

| Web of Science | | |
| --- | --- | --- |
| # | Query | Results |
| #1 | TS=("syndrome stein leventhal" OR "Stein Leventhal syndrome" OR "stein leventhal disease" OR "stein cohen leventhal syndrome" OR "Sclerocystic Ovary Syndrome" OR "Sclerocystic Ovary" OR "Sclerocystic Ovaries" OR "Sclerocystic Ovarian Degeneration" OR "polycystic ovary syndrome" OR "polycystic ovary disease" OR "polycystic ovary" OR "Polycystic Ovarian Syndrome" OR "polycystic ovarian disease" OR "ovary polycystic syndrome" OR "ovary polycystic disease" OR "multiple follicle cyst" OR "micropolycystic ovary" OR "cystic ovary") | 30786 |
| #2 | TS=("parental depression" OR "mental depression" OR "Emotional Depression" OR "depressivity" OR "depressive syndrome" OR "Depressive Symptoms" OR "depressive symptom" OR "depressive state" OR "depressive personality disorder" OR "depressive illness" OR "depressive episode" OR "depressive disorder" OR "depressive disease" OR "depression" OR "clinical depression" OR "central depression" OR "Social Anxiety" OR "Social Anxieties" OR "Nervousness" OR "Hypervigilance" OR "Anxiousness" OR "anxiety" OR "Angst") | 1019605 |
| #3 | #1 AND #2 | 1114 |
