# Supplementary material for: A network meta-analysis of interventions for anxiety and depression in PCOS
Source: PeerJ. 2026 Feb 5;14:e20744. doi: 10.7717/peerj.20744 (PMC12883158; doi:10.7717/peerj.20744)
Supplement: Supplemental Information 13 [file peerj-14-20744-s013.docx]

Table S2: Excluded Studies and Reasons After Reviewing the Full Text

| No | Author | Year | Title | Reasons for exclusion |
| --- | --- | --- | --- | --- |
| 1 | Alesi | 2022 | Nutritional Supplements and Complementary Therapies in Polycystic Ovary Syndrome | 1 |
| 2 | Kataoka | 2024 | Effects of weight loss intervention on anxiety, depression and quality of life in women with severe obesity and polycystic ovary syndrome | 2 |
| 3 | Wayadande | 2023 | Effectiveness of Callisthenic Exercises on BMI, Waist-Hip Ratio, Depression and Quality of Life in Class 1 Obese Young Adult Girls with Poly Cystic Ovarian Syndrome: An Experimental Study | 2 |
| 4 | Dema | 2023 | Effects of Mindfulness-Based Therapy on Clinical Symptoms and DNA Methylation in Patients with Polycystic Ovary Syndrome and High Metabolic Risk | 2 |
| 5 | Dilek | 2022 | Impact of the individual counseling program for polycystic ovarya syndrome management among university students: A prospective randomized controlled trial | 2 |
| 6 | Alkoudsi | 2020 | Assessing the effectiveness of a pharmaceutical care service on the quality of life of women with polycystic ovarian syndrome living in war and non-war countries | 2 |
| 7 | Amiri | 2020 | Effects of oral contraceptives on the quality of life of women with polycystic ovary syndrome: a crossover randomized controlled trial | 2 |
| 8 | Jiskoot | 2020 | Long-term effects of a three-component lifestyle intervention on emotional well-being in women with Polycystic Ovary Syndrome (PCOS): A secondary analysis of a randomized controlled trial | 2 |
| 9 | Abdollahi | 2019 | Effectiveness of cognitive-behavioral therapy (CBT) in improving the quality of life and psychological fatigue in women with polycystic ovarian syndrome: a randomized controlled clinical trial | 2 |
| 10 | Altinok | 2018 | Effect of 12-month treatment with metformin and/or oral contraceptives on health-related quality of life in polycystic ovary syndrome | 2 |
| 11 | Arentz | 2017 | Combined Lifestyle and Herbal Medicine in Overweight Women with Polycystic Ovary Syndrome (PCOS): A Randomized Controlled Trial | 2 |
| 12 | Kashani | 2013 | Does pioglitazone improve depression through insulin-sensitization? Results of a randomized double-blind metformin-controlled trial in patients with polycystic ovarian syndrome and comorbid depression | 2 |
| 13 | Foscolou | 2024 | Optimizing Dietary Habits in Adolescents with Polycystic Ovary Syndrome: Personalized Mediterranean Diet Intervention via Clinical Decision Support System-A Randomized Controlled Trial | 3 |
| 14 | Weiss | 2021 | Lifestyle modifications alone or combined with hormonal contraceptives improve sexual dysfunction in women with polycystic ovary syndrome | 3 |
| 15 | Alkoudsi | 2020 | Prevalence of anxiety and depression among women with Polycystic Ovary Syndrome living in war versus non-war zone countries: A randomized controlled trial assessing a pharmacist intervention | 3 |
| 16 | Oberg | 2020 | Psychological well-being and personality in relation to weight loss following behavioral modification intervention in obese women with polycystic ovary syndrome: a randomized controlled trial | 3 |
| 17 | Thomson | 2016 | Perceived exercise barriers are reduced and benefits are improved with lifestyle modification in overweight and obese women with polycystic ovary syndrome: a randomised controlled trial | 3 |
| 18 | Glintborg | 2015 | Increased thrombin generation in women with polycystic ovary syndrome: A pilot study on the effect of metformin and oral contraceptives | 3 |
| 19 | Thomson | 2010 | Lifestyle management improves quality of life and depression in overweight and obese women with polycystic ovary syndrome | 3 |
| 20 | Galletly | 2007 | Psychological benefits of a high-protein, low-carbohydrate diet in obese women with polycystic ovary syndrome--a pilot study | 3 |
| 21 | Clayton | 2005 | A randomized controlled trial of laser treatment among hirsute women with polycystic ovary syndrome | 3 |
| 22 | Wely | 2004 | Laparoscopic electrocautery of the ovaries versus recombinant FSH in clomiphene citrate-resistant polycystic ovary syndrome. Impact on women's health-related quality of life | 3 |
| 23 | Yin | 2021 | Can Psychosocial Intervention Suppress Testosterone and Triglycerides Among Women With Polycystic Ovary Syndrome? A Feasibility Trial | 4 |
| 24 | Cooney | 2018 | Cognitive-behavioral therapy improves weight loss and quality of life in women with polycystic ovary syndrome: a pilot randomized clinical trial | 4 |
| 25 | Vizza | 2016 | The feasibility of progressive resistance training in women with polycystic ovary syndrome: a pilot randomized controlled trial | 4 |
| 26 | Kogure | 2020 | The effects of aerobic physical exercises on body image among women with polycystic ovary syndrome | 5 |
| 1.Review;2.No anxiety/depression indicators,OR pre and post-data changes could not be extracted;3.Interventions unclear OR unable to link to the network structure;4.Sample size is too small (less than 20);5.The study sample overlaps with the included Lopes (2018), PMID: 30316737. | | | | |
