# Supplementary material for: A network meta-analysis of interventions for anxiety and depression in PCOS
Source: PeerJ. 2026 Feb 5;14:e20744. doi: 10.7717/peerj.20744 (PMC12883158; doi:10.7717/peerj.20744)
Supplement: Supplemental Information 14 [file peerj-14-20744-s014.docx]

## Systematic Review and/or Meta-Analysis Rationale

For systematic reviews / meta-analyses, authors need to provide the following information:

1.The rationale for conducting the systematic review / meta-analysis.

**Reply:** The aim of this study is to provide evidence for the best interventions to improve anxiety and depression in patients with polycystic ovary syndrome through a systematic review and network meta-analysis.

2.The contribution that it makes to knowledge in light of previously published related reports, including other meta-analyses and systematic reviews.

**Reply:** Unlike traditional direct comparison methods, network meta-analysis establishes the relative effects between multiple treatment methods through indirect evidence, allowing for a more comprehensive comparison and evaluation of the effectiveness of different interventions in improving anxiety and depression in patients with polycystic ovary syndrome. This analytical approach helps provide more scientific and high-quality evidence to support clinical decision-making, guiding doctors in selecting the most appropriate treatment plan based on the specific conditions of the patients.
